# Auditory Electrooculogram-based Communication System for ALS Patients in Transition from Locked-in to Complete Locked-in State

**DOI:** 10.1038/s41598-020-65333-1

**Published:** 2020-05-21

**Authors:** Alessandro Tonin, Andres Jaramillo-Gonzalez, Aygul Rana, Majid Khalili-Ardali, Niels Birbaumer, Ujwal Chaudhary

**Affiliations:** 1 Institute of Medical Psychology and Behavioral Neurobiology, University of Tübingen, Tübingen, Germany; 2 Wyss-Center for Bio- and Neuro-Engineering, Geneva, Switzerland

**Keywords:** Translational research, Amyotrophic lateral sclerosis

## Abstract

Patients in the transition from locked-in (i.e., a state of almost complete paralysis with voluntary eye movement control, eye blinks or twitches of face muscles, and preserved consciousness) to complete locked-in state (i.e., total paralysis including paralysis of eye-muscles and loss of gaze-fixation, combined with preserved consciousness) are left without any means of communication. An auditory communication system based on electrooculogram (EOG) was developed to enable such patients to communicate. Four amyotrophic lateral sclerosis patients in transition from locked-in state to completely locked-in state, with ALSFRS-R score of 0, unable to use eye trackers for communication, learned to use an auditory EOG-based communication system. The patients, with eye-movement amplitude between the range of ±200μV and ±40μV, were able to form complete sentences and communicate independently and freely, selecting letters from an auditory speller system. A follow-up of one year with one patient shows the feasibility of the proposed system in long-term use and the correlation between speller performance and eye-movement decay. The results of the auditory speller system have the potential to provide a means of communication to patient populations without gaze fixation ability and with low eye-movement amplitude range.

## Introduction

Swiss philosopher Ludwig Hohl stated that “The Human being lives according to its capacity to communicate, losing communication means losing life”^1^. Our ability to communicate ideas, thoughts, desires, and emotions shapes and ensures our existence in a social environment. There are several neuronal disorders, such as amyotrophic lateral sclerosis (ALS), or brain stem stroke, among others, which paralyzes the affected individuals severely impairing their communication capacity^2–5^. The affected paralyzed individuals with intact consciousness, voluntary eye movement control, eye blinks, or twitches of other muscles are said to be in locked-in state (LIS)^6–11^.

Early and modern descriptions of ALS disease emphasize that oculomotor functions are either spared or resistant to the progression of the disease^9^, and consequently, eye-tracking devices can be used to enable patients in the advanced state of ALS to communicate^12,13^. Besides, longitudinal studies evaluating eye-tracking as a tool for cognitive assessment report that the progression of the disease does not affect eye-tracking performance^9^. Nevertheless, a subset of the literature reports a wide range of oculomotor dysfunctions in these patients^14–17^ that might prevent the use of eye-tracking devices^18^. The most used metric to evaluate the patient’s degree of functional impairment is the revised ALS functional rating scale (ALSFRS-R)^19^, which is not a precise measure of the ability to communicate. A patient with an ALSFRS-R score of zero can still have eye-movement capability or control over some other muscles of the body, which can be used for communication^8^.

CLIS is an extreme type of LIS, which leads to complete body paralysis, including paralysis of eye-muscles combined with preserved consciousness^6,20^; therefore, even if the individuals are incapable of voluntary control of any muscular channels of the body, they might remain cognitively intact^9^. Several systemic or traumatic neurological diseases may result in a LIS with the potential to progress towards CLIS, such as ALS, Guillain-Barré, pontine stroke, end-stage Parkinson disease, multiple sclerosis, traumatic brain injury and others with different etiological and neuropathological features^4,7,10^. In the case of ALS patients in LIS who survive longer attached to life-support systems, the disease progression might ultimately destroy the oculomotor control in many patients, leading to the loss of gaze-fixation^17^. Thus, patients become unable to use eye-tracker-based communication technologies and are therefore left without any means of communication. This raises the question, what happens with those ALS patients in transition from LIS to CLIS with highly compromised oculomotor skills unable to retain gaze-fixation, and therefore unable to use eye-tracking systems to communicate?

There is a considerable amount of research related to patients in the early stages of ALS who can successfully achieve communication by using gaze-fixation-based assistive and augmentative communication (AAC) technologies or brain-computer interfaces (BCIs). These patients have intact cognitive skills, residual voluntary movements, intact or partial vision with complete gaze-fixation capabilities. Several examples of communication technologies for ALS patients in LIS can be found in the literature. Concerning BCI-based communication, different types of systems have been developed to provide a means of communication to LIS patients^4,10,11^, among the most recognized are the ones based in features of the EEG, as the slow cortical potential^21^, or evoked potentials, mainly the P300^22–26^ or SSVEP^27^; or the BCIs based in metabolic features, as NIRS^28–31^. Concerning the use of eye-tracking systems, as long as the patients have intact vision and control gaze fixation, commercial systems are an accessible and reliable option to allow them limited communication^32^. Other types of eye-tracking technologies as the scleral search soils, infrared reflection oculography, or video-oculography (or video-based eye-tracking)^33^, have not yet been tested on LIS patients to our knowledge. Except for two studies^28,29^, all the developed BCIs for ALS patients describe patients with remnant muscular activity, remnant eye movement control, or even without assisted ventilation, and in general with ALSFSR-R score above 15.

The progress of ALS often, if not always, diminishes the general capabilities of the patients making BCI-based communication impossible^7,24^. On the other hand, even though eye movement might be the last remnant voluntary movement before CLIS^34^, during this transitional state from LIS to CLIS, patients become unable to maintain gaze-fixation and, unable to use eye-tracking AAC technologies.

Some electrooculogram (EOG) -based systems have been presented to overcome the limitations of other AAC technologies. However, most of the studies were performed on healthy participants or tested in LIS patients in the early stages of ALS, reporting results of single sessions or sessions performed closely in time, allowing the patients in advanced LIS to reply yes/no questions, but without the feasibility of freely communicate spelling sentences^35,36^. To our knowledge, no studies report on the long-term use of EOG or eye-tracking for patients on the transition from LIS to CLIS, and how this progression affects communication capabilities using these AAC technologies. Either in clinical descriptions or technical applications, very little is known about how this LIS to CLIS progression affects the oculomotor capabilities precluding the patient’s communication.

Considering ALS patients in an advanced state, a first meta-analysis has shown that there is a correlation between the progression of physical impairment and BCI performance^7^, and a recent one has suggested that the performance of CLIS patients using BCI cannot be differentiated from chance^37^. The only available long-term studies are either single cases for patients able to perform with a P300-based BCI^38,39^ or a thoroughly home-based BCI longitudinal study^40^ that shows favorable results. However, these studies do not provide details on how the progression affected the performance, particularly for the patients with the lowest ALSFSR-R score.

It has been shown in a single case report^34^, that during the transition from LIS to CLIS, despite compromised vision due to the dryness and necrosis of the cornea and inability to fixate, some remaining controllable muscles of the eyes continue to function. Hence, there is an opportunity to develop a technology to provide a means of communication in this critical transition. Such a technology would extend these patients’ communication capacities until the point the disease progression destroys any volitional motor control. Pursuing that goal, an EOG-based auditory communication system was developed, which enabled patients to communicate independent of their gaze fixation ability and independent of intact vision. This study was performed with four ALS patients in transition from the locked-in state to the completely locked-in state, with ALSFRS-R score of 0, and unable to use eye-trackers effectively for communication, i.e., without any other means of communication. The patients, with eye-movement amplitude between the range of ±200 μV and ±40 μV, were able to form complete sentences and communicate independently and freely, selecting letters from an auditory speller system. Moreover, the study shows the possibility of using the proposed system for a long-term period, and, for one patient, it shows the decay in the oculomotor control, as reflected in EOG signals, until the complete loss of eye control. Such a communication device will have a significant positive effect on the quality of life of completely paralyzed patients and improve mandatory 24-hours-care.

## Results

Four advanced ALS patients (P11, P13, P15, and P16) in the transition from LIS to CLIS, all native German speakers (Table 1), used the developed auditory communication system to select letters to form words and hence sentences. All the patients attended to four different types of auditory sessions: training, feedback, copy spelling, and free spelling session. Each training and feedback sessions consisted of 20 questions with known answers (10 questions with “yes” answer and 10 questions with “no” answer, presented in random order), for example, “Berlin is the capital of Germany” vs. “Paris is the capital of Germany”. All the questions were presented auditorily. While in the copy and free spelling sessions, the patients were presented the group of characters and each character auditorily (see “Methods“ section for the details). Patients were instructed to move the eyes (“eye-movement”) to say “yes” and not to move the eyes (“no eye-movement”) to say “no”. Features of the EOG signal corresponding to “eye-movement” and “no eye-movement” or “yes” and “no” were extracted to train a binary support vector machine (SVM) to identify “yes” and “no” response. This “yes” and “no” response was then used by the patient to auditorily select letters to form words and hence sentences during the feedback and spelling sessions. Due to the degradation of vision in ALS patients^14,16,17^, the system was designed to work only in the auditory mode without any video support. We frequently traveled to the patient’s home to perform the communication sessions. Each visit (V) lasted for a few days (D), during which the patient performed different session (S) as listed in Supplementary Tables S1–S4.

**Table 1:** List of participants.

Patient	Gender/Age	ALS type	Medical history	Visits
P11	M/33	Non-bulbar	Aug 2015: Diagnosis	10 visits over 13 months from March 2018 onwards
			Aug 2017: Last use of AAC	
P13	M/58	Bulbar	Jan 2011: Diagnosis	4 visits over 12 months from Jun 2018 onwards
			Jan 2018: Last use of AAC	
P15	F/63	Lower motor neuron predominant (ICD-10: G12.2)	Feb 2017: Diagnosis	2 visits over 5 months from Feb 2019 onwards
			Nov 2018: Last use of AAC	
P16	M/56	Lower motor neuron	Dec 2012: Diagnosis	2 visits over 3 months from March 2019 onwards
			Jun 2018: Last use of AAC	

The table lists the patient’s number, the gender and the age at the time of the first visit, the type of diagnosed ALS, year of diagnosis, and the last use of assistive and augmentative communication (AAC) technologies, and the number of performed visits and their time range.

### Eye movement

**Figure 1:**
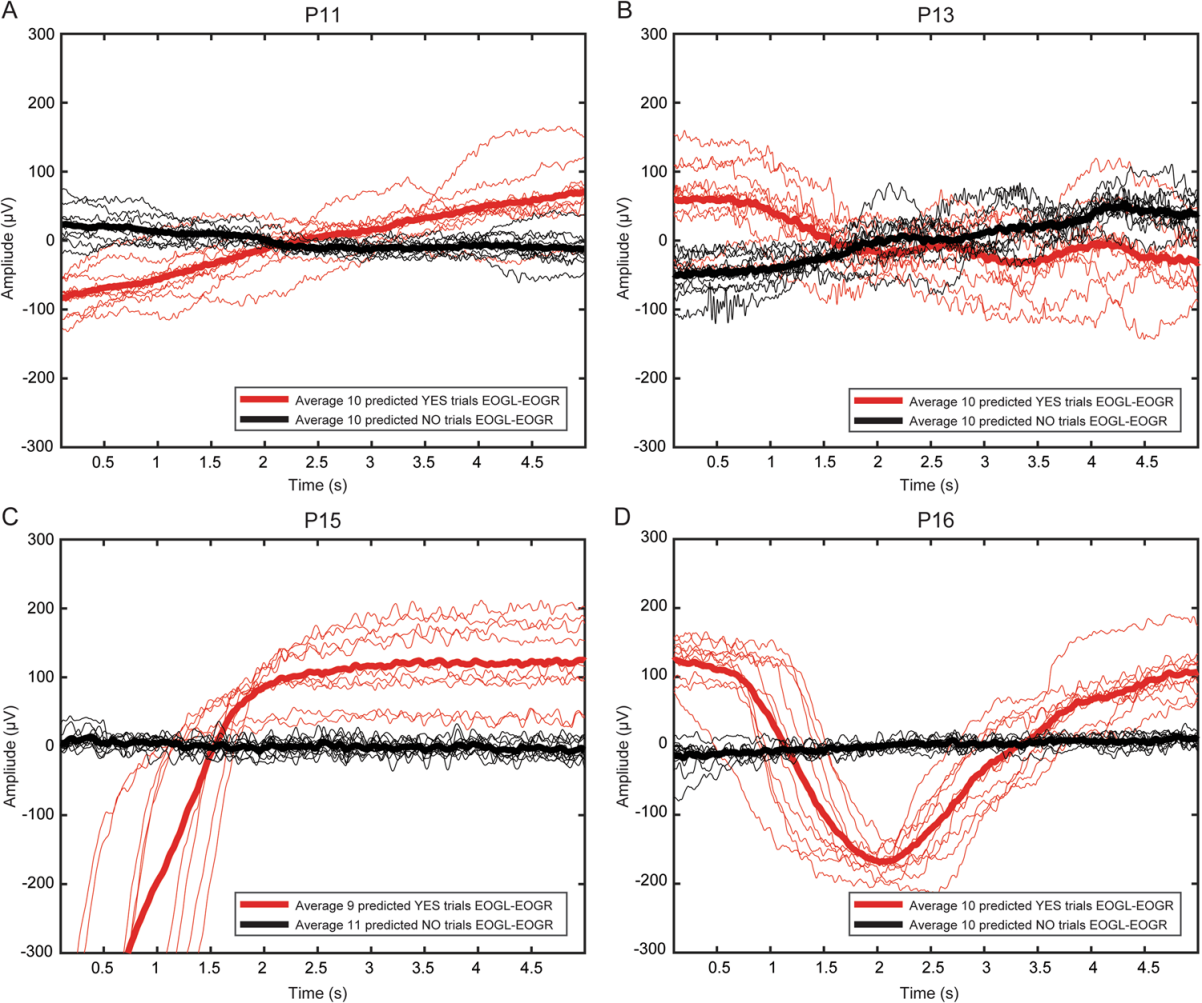
Horizontal eye movement during feedback sessions for all patients. Differential channel EOGL-EOGR for a particular feedback session performed by (**A**) P11, (**B**) P13, (**C**) P15, and (**D**) P16 during the first visit. In each subfigure, the x-axis is the response time in seconds, and the y-axis is the amplitude of the eye-movement in microvolts (μV). The thin and thick red trace corresponds to a single “yes” response and average of all the “yes” responses, respectively. The black thin and thick trace corresponds to a single “no” response and average of all the “no” response. The box at the bottom right of each subfigure lists the number of trials classified as “yes” and “no” by the SVM classifier for that particular session.

According to the literature, in healthy subjects, the amplitude of the EOG signal varies from 50 to 3500 µV, and its behavior is practically linear for gaze angles of ±30 degrees and changes approximately 20 µV for each degree of eye movement^41,42^. Nevertheless, like any other biopotential, EOG is rarely deterministic; its behavior might vary due to physiological and instrumental factors^32^. For LIS patients in the transition to CLIS, the range and angle of movement are affected by the progress of the ALS disease, affecting the range of voltage amplitude as well. Figure 1 depicts the horizontal eye movement of P11, P13, P15, and P16 during one of the feedback sessions of their first visit (V01). In each plot, for a particular feedback session, all the questions’ responses classified as “yes” or “no” by the SVM models were grouped and averaged. Figure 1 elucidates the differences in the dynamics of the signals corresponding to the “yes” and “no” responses, and it can be observed that each patient used different dynamics to control the auditory communication system.

**Figure 2:**
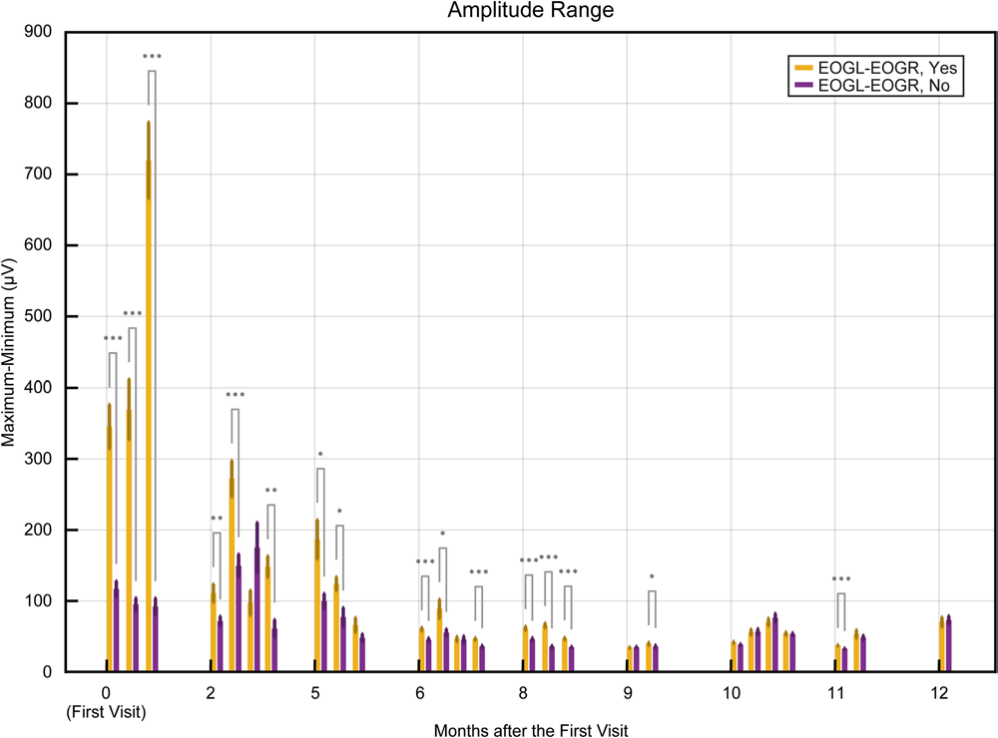
Progressive decline of the eye-movement amplitude along the visits for P11. Depicts the trend of decline in the range of the amplitude of the EOG signal for yes/no questions answered by the patient during the period March 2018 to March 2019. The figure shows the mean and the standard error of the mean of the extracted range of the amplitude of the horizontal EOG signal across each day for yes and no trials. The x-axis represents the month of the sessions, and the y-axis represents the amplitude in microvolts. The asterisk (* - p-value less than 0.05; ** - p-value less than 0.01; *** - p-value less than 0.001) in the figure represents the results of the significance test performed between yes and no for horizontal EOG employing the Mann-Whitney U-test.

Figure 2 depicts a decrease in horizontal eye-movement amplitude of P11 over 13 months. During the 12 months period, from March 2018 (V01) to February 2019 (V09), P11 performed feedback sessions with a prediction accuracy above chance level, in which small eye-movements recorded with EOG allow classification of “yes” and “no” signals. Employing the same eye-movement dynamics with an approximate amplitude range smaller than ±40 µV over 4 months, from V06 (November 2018) to V09 (February 2019), P11 was able to select letters, words and form sentences using the speller. The eye-movement amplitude range decreased to ±30 µV during V10, i.e., 12 months after the first BCI sessions, because of the progressive paralysis typical of ALS. During V10, model-building for prediction during feedback and spelling sessions was unsuccessful. Thus, V10 was the last visit for a communication attempt by P11 using this paradigm. During this visit, even if this training session allowed to build a model of 80% of cross-validation accuracy (Supplementary Table S1), it proved unsuccessful for predicting any classes from the data (50% accuracy).

In the case of P13, the progression of the disorder has been slower, which can be ascertained by the relatively high and constant amplitude of EOG, in an approximate range of ±300 µV, but still, he was unable to communicate with the commercial eye-tracker technology. Employing the eye-movement strategy, as shown in Fig. 1B, P13 was able to maintain a constant dynamic to control the auditory communication system for feedback and spelling sessions (see Supplementary Table S2). Similar observations can be drawn for P15 and P16 EOG plots in Fig. 1C,D. During two visits each, they achieved successful performance for feedback and spelling sessions (see Supplementary Tables S3 and S4), with stable eye-movement dynamics.

### Speller results

**Figure 3:**
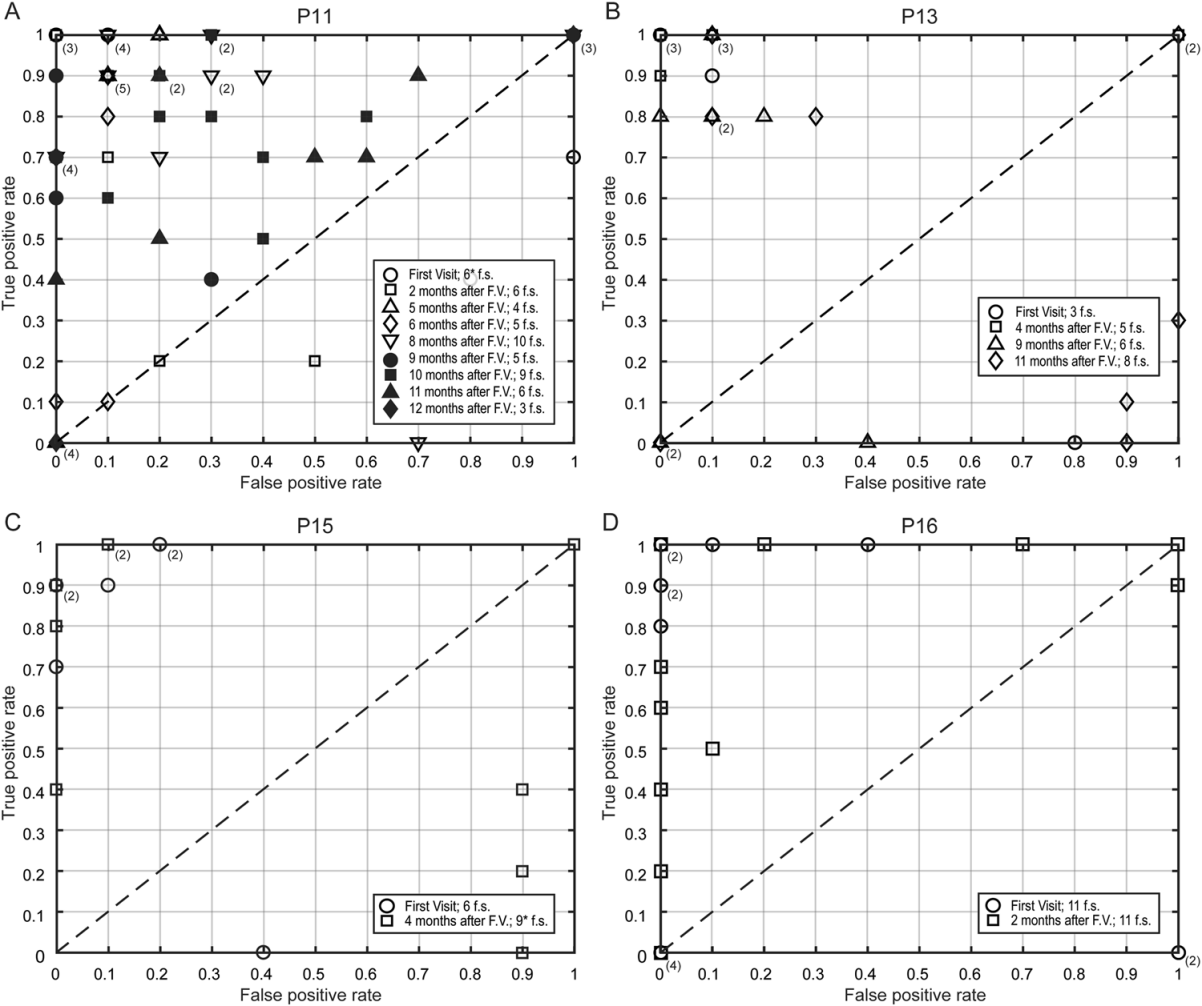
ROC space of feedback sessions for the four patients. Receiver operating characteristic (ROC) space for the performance of the binary support vector machine (SVM) classifier during the total number of feedback sessions performed by (A) P11, (B) P13, (C) P15, and (D) P16. In the figures, the x-axis is the false positive rate (FPR), and the y-axis is the true positive rate (TPR). The diagonal line dividing the ROC space represents a 50% level. Points above the diagonal represent good classification results (accuracy better than 50%), points below the line represent poor classification results (accuracy worse than 50%). In each subfigure, FPR vs. TPR for the feedback sessions are indicated by different arbitrary symbols according to the visit (V) they belong and the date, as defined in the legend at the bottom right side of each subfigure. The rectangular box at the bottom right of each subfigure lists the visit’s month and the number of feedback sessions performed during each visit. Some feedback sessions have the same coordinate values in the ROC space, and their symbols overlapped; in these cases, the number of overlapped symbols is specified in parenthesis close to the symbols.

The performance of the SVM during all the feedback sessions by each patient is reported in Fig. 3 as a Receiver-Operating Characteristic (ROC) space. The ROC space of P11, who was followed for one year from March 2018 to March 2019, shows a trend in the performance of the feedback sessions. As shown in Fig. 3A, during the initial visits P11 exhibited a successful feedback performance (markers located in the upper-left corner in the ROC space), while during the later visits, particularly V08 and V09, P11 exhibited a decrease in feedback performance and ultimately by V10, it was impossible to perform a successful feedback session. This negative trend is due to the progressive neurodegeneration associated with ALS^8^, which leads to the complete paralysis of all muscles, including eyes muscles. For each of the three patients P13, P15, and P16, the feedback sessions’ performances are located mostly in the upper-left region of the ROC space, which means successful feedback performance. Nevertheless, for each of these three patients, a few feedback sessions also fall in the lower-right region of the ROC space. This might be due to a learning process of the patients in which they improved or adjusted their eye-movement strategy or due to the suboptimal performance of the SVM classifier during the first few feedback sessions.

The patients were asked to attempt a spelling session when a model was validated with a successful feedback session, i.e., results above random^43^. After the feedback session, patients performed two different types of spelling sessions: copy spelling and free spelling, i.e., sessions in which the patient was asked to spell a predetermined phrase, and sessions in which the patient spelled the sentence she/he desired.

In the developed auditory communication system, the letters have been grouped in different sectors in a layout that was personalized for each patient to match the paper-based layout developed independently by each family (Supplementary Fig. S1). To select one letter, every sector was sequentially presented to the patient and the patient auditorily selected or skipped a sector, once a sector was selected the letters inside the sector were presented auditorily. This select/skip paradigm (i.e., yes/no answer to auditory stimuli) allows the system to work using just a binary yes/no response. The patient could form words by selecting every single letter, but the speed of the system was improved by a word predictor, which, based on the previous selections, suggested the completion of a word whenever it was probable. The speller algorithm is described in detail in the section Speller algorithm.

The results of the copy spelling sessions performed by all the patients are reported in Supplementary Table S5. As shown in Table 2, P11 performed 14 copy-spelling sessions out of which 7 times he correctly copy-spelled the target phrase. Moreover, in one of the other cases, he just miss-selected one letter, and in another one, he selected only one of the two requested words. P13 over 8 sessions copy spelled correctly the target word 6 times. P15 selected correctly the target phrase 3 times out of 5 sessions. Finally, P16 was able to correctly copy spell a target phrase 3 out of 5 sessions. The typing speed achieved by each patient is shown in Table 2.

**Table 2:** Results of the spelling sessions performed by the four patients.

Type	Patient	Number of sessions	Characters selected	Speed (char/min)
Copy	P11	7/14	5,28 ± 3,59	0,54 ± 0,30
	P13	6/8	4,00 ± 1,67	0,50 ± 0,35
	P15	3/5	5,00 ± 0,00	0,49 ± 0,30
	P16	3/5	5,00 ± 1,73	0,69 ± 0,14
Free	P11	5/9	11,60 ± 8,79	0,57 ± 0,29
	P13	10/11	13,00 ± 10,34	0,48 ± 0,24
	P15	4/5	26,67 ± 19,14	0,68 ± 0,13
	P16	3/3	14,00 ± 4,36	0,64 ± 0,13

The columns indicate the type of sessions, the patient, the number of considered sessions over the total number of sessions, the number of characters selected (mean ± standard deviation), and the typing speed in characters per minute (mean ± standard deviation). For the copy and free spelling sessions, only the sessions in which the target was spelled correctly, and the spelled sentence was meaningful, respectively, have been considered. Sessions have been excluded a priori if an error in the code occurred, if the signal was noisy, if they terminated before 15 trials, if the patient did not select any letter, or if all the answers have been classified only as “yes” or only as “no”: in total 6 sessions from P11, 2 sessions from P13, 10 sessions from P15, and 3 sessions from P16 were excluded.

The system can present one question every 9 seconds, which implies an information transfer rate of 6.7 bits/min. The optimal speed of the speller, along with the user accuracy, depends on the two factors mentioned: first, the speller design for the letter selection (Supplementary Fig. S1); second, the collection of stored sentences (i.e., corpus) needed for the word prediction. In order to describe and evaluate the performance, the sentence “Ich bin” (German for “I am”) followed by the name of the patient was considered as a standard example for P11 and P13, while the name of the spouse was considered for P15 and P16. These standard example sentences are composed of 13, 11, 5, and 6 characters for P11, P13, P15, and P16, respectively. Therefore, considering no errors in the answers’ classification, the average typing speed for the sentence mentioned above is 1.14 char/min for P11, 1.19 char/min for P13, 1.08 char/min for P15, and 0.87 char/min for P16. These theoretical results show that, due to the word prediction, the performance of the speller improves when the patient auditorily spells a complete sentence rather than a single word. The difference between the theoretical and the real typing speed is due to the nature of the speller that requires many inputs to correct a mistake, e.g., if a sector is wrongly skipped, to select that sector again the patient must first skip all the other sectors.

After successful copy spelling sessions, the patients were free to form words and sentences of their choice. The results of these free spelling sessions performed by all the patients are reported in Supplementary Table S6. The typing speed in these sessions is similar to the speed achieved by each patient during the copy spelling; one exception is P13 who due to the low number of errors and to better words’ prediction reached the speed of 1.02 char/min during one of the sessions shown in Supplementary Table S6. In most of the sessions, the patients were able to form complete sentences communicating their feelings and their needs. Nonetheless, some of the performed sessions were not successful. Videos of selected spelling sessions are available in Supplementary Videos S1–S3.

## Discussion

The auditory communication system enabled four ALS patients, with ALSFRS-R score of 0, on the verge to CLIS to select letters and words to form sentences. Three out of the four patients (P13, P15, and P16) showed, during all the sessions, a preserved eye movement. One patient (P11), followed over one year from March 2018 to March 2019, demonstrated an effective eye-movement control until the penultimate visit (V09 in February 2019), despite August 2017 being the last successful communication with a commercial AAC device. However, the progression of the disease varies from patient to patient.

Nonetheless, P11’s successful results of V09, even if not perfect, are very encouraging since they show the possibility of communicating even with an eye-movement amplitude range of ±30 µV. Even if the developed auditory communication system was used only from V06, the evolution of eye movements of P11 (Fig. 2) indicates that the eye signal was clear enough to be used for communication purposes since the first visit in March 2018. The results of the feedback sessions confirm this during the initial five visits (Fig. 3A). Speed is the main limitation of the developed system since the spelling of one single word could take up to 10 minutes. In the literature, other spelling systems have been successfully tested with ALS patients, and they achieved an information transfer rate of 16.2 bits/min^44^ and 19.95 bits/min^45^. However, since all of them are based on visual paradigms, except a single study where the patients communicated just “yes” or “no” using an auditory system^46^, comparison with the here proposed system is difficult. The slow speed of our system is an intrinsic characteristic of its auditory nature, even though the spelling time can be reduced by optimizing the speller schema and improving the word prediction with the creation of a corpus of words personalized for each patient. Even though the user experience was not assessed with a questionnaire, it is vital to notice that the patients showed no frustration for this slow speed, which they indicated by moving their eyes when questioned, “Would you like to continue?”. The patient formed sentences like, “I am Happy”, “I am happy to see my grandchildren growing up”, and “I look forward to a vacation” indicating their willingness to communicate. From these, we infer speculatively that the slow speed did not frustrate the patients, probably because even this slow communication is preferred and valued in comparison to the isolation experienced without a functioning eye-tracker. It is essential to employ such a paradigm and follow these patients regularly to elucidate their eye-movement dynamics further and provide them a means of communication.

In conclusion, the long-term viability of an EOG based auditory speller system in ALS patients on the verge of CLIS (with ALSFSR-R score of 0) unable to use eye-tracking based AAC technologies were explored. For one of the patients, it was possible to perform a long-term recording, capturing the changes in the EOG signal, evidencing a correlation between speller performance and progressive degeneration of the oculomotor control. After a follow-up of one year, the patient was unable to take advantage of the spelling system proposed because of the complete loss of oculomotor control. Although the reported system cannot be considered as the ultimate communication solution for these patients according to the best of the authors’ knowledge, this is the only system that, during the period of transition from LIS to CLIS, might offer a means of communication that otherwise is not possible. Nevertheless, whether this can be generalized to other patient populations or not is an empirical question.

## Methods

The Internal Review Board of the Medical Faculty of the University of Tubingen approved the experiment reported in this study. The study was performed per the guideline established by the Medical Faculty of the University of Tubingen. The patient or the patients’ legal representative gave informed consent with permission to publish the results and to publish videos and pictures of patients. The clinical trial registration number is ClinicalTrials.gov Identifier: NCT02980380.

### Instrumentation

During all the sessions, EOG channels were recorded with a 16 channel EEG amplifier (V-Amp DC, Brain Products, Germany) with Ag/AgCl active electrodes. A total of four EOG electrodes were recorded (positions SO1 and IO1 for vertical eye movement, and LO1 and LO2 for horizontal eye movement). During some sessions, a minimum of seven EEG channels were recorded for analysis, not directly related to the purpose of this paper. All the channels were referenced to an electrode on the right mastoid and grounded to the electrode placed at the FPz location on the scalp. For the montage, electrode impedances were kept below 10 kΩ. The sampling frequency was 500 Hz.

### Patients

Four ALS patients with ALSFRS-R score of 0 participated in this study. Table 1 summarizes the clinical history of each patient and lists the number of visits (V). After the last successful use of AAC, all the four patients were still communicating with the relatives saying “yes” and “no” by moving and not moving the eyes. Using this technique, patients P11, P13, and P15 were forming words by selecting letters from a paper-based layout (Supplementary Fig. S1A–C) developed, independently, by each family. These same layouts were integrated into our developed system to provide each patient with a personalized schema for selecting letters. For patient P16, based on the feedback and suggestions of the family members, we proposed and tested the spelling schema shown in Supplementary Fig. S1D.

### Paradigm

The developed paradigm is based on a binary system, in which a patient is asked to reply to an auditorily presented question by moving the eyes to say “yes” and by not moving the eyes to say “no”. The paradigm includes four different types of sessions: training, feedback, copy spelling, and free spelling session. Each training and feedback session consists of 10 questions with a “yes” answer and 10 questions with a “no” answer well known by the patient. Each question represents a trial. Copy and free spelling sessions consist of yes/no questions (i.e., trial) in which a patient is asked whether he wants to select a particular letter or group of letters (see the below paragraph Speller algorithm). Each of these trials consists of the baseline (i.e., no sound presented), the stimulus (i.e., auditory presentation of the question and the speller options), the response time (i.e., time for the patient to move or not move the eyes), and feedback (i.e., auditory feedback to the patient to indicate the end of the response time). The training sessions differ from the feedback sessions in terms of the feedback that is provided to the patient. During the training sessions, the feedback is a neutral stimulus (“Danke” – “Thank you” in English) to indicate the end of the response time, while during the feedback sessions, the feedback is the answer that the program classifies (see Online analysis for details). Copy and free spelling sessions differ in terms of the instruction given to the patient. During the copy spelling sessions, the patient was asked to spell a specific sentence, while during the free spelling sessions, the patient was asked to spell whatever he desired.

The length of the response time-window was determined according to the progress and performance of the patient as described in the Supplementary Tables S1–S4 and varies between 3 and 10 seconds. The duration of each trial varies accordingly between 9 and 20 seconds. Therefore, each training and feedback session lasted for 3–7 minutes. The spelling sessions were usually longer (up to 57 minutes), but no fixed time can be indicated since the number of trials is different from session to session; on average, the copy and free spelling sessions lasted respectively for 10 and 27 minutes.

### Speller algorithm

After the patients were unable to communicate with the commercial eye-tracker based AACs devices, the primary caretakers developed a speller design/layout. The auditory speller layouts used here were developed by optimizing and automatizing the schematics already used by the primary caretakers. The spellers used by the patients are shown in Supplementary Fig. S1. The spellers consist of letters grouped in different sectors, plus one sector with some special characters (“space”, “backspace” for P11 and P15, and for P13 and P16 along with these the additional option, “delete the word”). Despite the different layouts, the same algorithm, as described below, drives all the spellers. The spellers enable the patients to auditorily select letters and compose words. To increase the speed of the sentence completion, the speller predicts and proposes words based on the letters previously chosen. The auditory speller developed to enable patients without any means of communication to spell letters, words freely, and form sentences auditorily have two main components called “Letter selection” and “Word prediction”, which are described below.

#### Letter selection

The patient, in order to select a letter, first must select the corresponding sector, and only once he is inside the sector, he can select the letter. The selection is made, answering “yes” or “no” to the auditory presentation of a sector or a letter. As schematized in the diagram in Supplementary Fig. S2, to avoid false positives, the speller uses a single-no/double-yes strategy. If the recognized answer is “no” the sector is not selected, and the following sector will be asked, if the answer is “yes” the same sector is asked a second time as a confirmation: the sector is selected if the patient replies “yes” also the second time. If the last sector is not selected, the program asks the patient whether he wants to quit the program. If he replies “yes”, and confirms the answer, the program is quit.

Otherwise, the algorithm restarts from the first sector. Once a sector is selected, the paradigm for selecting a letter (or a special character) uses the same single-no/double-yes strategy as described above. If none of the letters in a sector is selected, the patient is asked to exit the sector. If he replies a confirmed “yes” the speller goes back asking the sectors starting from the one after the current, otherwise, if he replies “no”, the algorithm asks the letters of the current sector again starting from the first one. Whenever a letter or a special character is selected, the speller updates the current string and gives auditory feedback reading the words already completed (i.e., followed by a space) and spelling the last one if it is not complete. After every selected letter, the speller searches for probable words based on the current string (the details are explained in the paragraph below). If a word is probable, the program presents that word auditorily. Otherwise, it starts the letter selection algorithm from the first sector again.

#### Word prediction

To speed up the formulation of sentences, the speller is provided with a word predictor that compares the current string with a language corpus to find if there is any word that has a high probability of being the desired one. To have a complete and reliable vocabulary, the German general corpus of 10000 sentences compiled by the Leipzig University^47^ was used. Since the developed speller contains only English letters, firstly the corpus is normalized converting the special German graphemes (ä, ö, ü, ß) with their usual substitutions (ae, oe, ue, ss). Thus, a conditional frequency distribution (CFD) is created based on the n-gram analysis of the normalized corpus; for word prediction, we considered the frequencies of the single words (i.e., unigrams), of two consecutive words (i.e., bigrams), and three consecutive words (i.e., trigrams). Whenever a letter is added to the current string, the program returns the CFD of all the words starting with the current non-completed word (if the last word is complete, it considers all the possible words). For these words first, the frequency value is considered concerning the two previous words, i.e., trigram frequency. Then, if for the current string, there is any stored trigram in the corpus, the program considers only the last complete word and checks the bigram frequency. Finally, if it is not possible to find any bigram, it considers the overall frequency value of the single word, i.e., unigram frequency. Once all the frequency values of the words are stored, considered as trigrams, bigrams, or unigrams, the program establishes if any of these words are highly probable comparing their values to a predefined threshold. To predict words, we considered a word as probable if its frequency value is more significant than half of the sum of all the frequency values of the possible words. If a word is detected as probable, the speller, after a letter is selected, instead of restarting the algorithm from the first sector, proposes that word to the patient, and if a confirmed “yes” is answered it adds the word followed by a space to the current string.

### Online analysis

The EOG data were acquired online in real-time throughout all the sessions. During all the trials belonging to a session (except for the training sessions), the signal of the response time was processed in real-time to extract features to be fed to a classification algorithm for classifying the “yes” and “no” answers. Features computed from the trials of the training sessions were used to train an SVM classifier that was validated through 5-fold cross-validation. The obtained SVM classifier was used to classify feedback and speller sessions only if its accuracy was higher than the upper threshold of chance-level^43^.

To extract the features from the signal during the response time, the time-series were first preprocessed with a digital finite impulse response (FIR) filter in the passband of 0.1 to 35 Hz and with a notch filter at 50 Hz. The first 50 data points were removed to eliminate filtering-related transitory border effects at the beginning of the signal. Then all the channels were standardized to have a mean of zero and a standard deviation of one. Subsequently, features were extracted from all the data series from the “yes” and “no” answer for all the channels.

Different features were extracted for the different patients: for P11 and P13 the maximum and minimum amplitude and their respective value of time occurrence feature were used; while for P15 and P16 the range of the amplitude (i.e., the difference between the values of maximum and minimum amplitude) feature was used.

The code was developed and run in Matlab_R2017a. For the SVM classification, the library LibSVM^48^ was used. The detailed list of sessions used for building the model and, therefore, perform feedback and spelling sessions are described in the Supplementary Tables S1–S4.

### Receiver-operating characteristic space

For binary classifiers in which the result is only positive or negative, there are four possible outcomes. When the outcome of the prediction of the answer is yes (positive), and the actual value is positive, it is called True Positive (TP); however, if the actual answer to a positive question response is negative, then it is a False Negative (FN). Complementarily, when the predicted answer is negative, and the actual answer is also negative, this is a True Negative (TN), and if the prediction outcome is negative and the actual answer is positive, it is a False Negative (FN). With these values, it is possible to formulate a confusion or contingency matrix, which is useful to describe the performance of the classifier employing its tradeoffs between sensitivity and specificity. The contingency matrix can be used to derive several evaluation metrics, but it is particularly useful for describing and visualizing the performance of classifiers via the Receiver-Operating Characteristic (ROC) space^49^. A ROC space depicts the relationship between the True Positive Rate (TPR) and the False Positive Rate (FPR). TPR and FPR were calculated for each feedback session, and they were then used to draw ROC space, as shown in Fig. 3.

## Supplementary information

Supplementary Materials.

Supplementary Video S1.

Supplementary Video S2.

Supplementary Video S3.

## Data Availability

The data and the scripts are available without any restrictions. The correspondence between sessions and the corresponding raw files are listed in Supplementary Table S7. Data link: 10.5281/zenodo.3605395.
