# Supplementary material for: Auditory Electrooculogram-based Communication System for ALS Patients in Transition from Locked-in to Complete Locked-in State
Source: Sci Rep. 2020 May 21;10:8452. doi: 10.1038/s41598-020-65333-1 (PMC7242332; doi:10.1038/s41598-020-65333-1)
Supplement: Supplementary file 1 — Supplementary Materials. [file 41598_2020_65333_MOESM1_ESM.pdf]

Auditory Electrooculogram-based Communication System for ALS Patients in Transition from Locked-in to Complete Locked-in State

Alessandro Tonin[1+], Andres Jaramillo-Gonzalez[1+], Aygul Rana[1], Majid Khalili Ardali[1],

Niels Birbaumer[1,2], Ujwal Chaudhary[1,2*]

[1]Institute of Medical Psychology and Behavioral Neurobiology, University of Tübingen, Germany

[2]Wyss-Center for Bio- and Neuro-Engineering, Geneva, Switzerland

+These authors contributed equally

* Corresponding author: chaudharyujwal@gmail.com

Supplementary Figure S1:

Schema of the speller designed for the four patients.

Different schemas used for (A) P11, (B) P13, (C) P15, and (D) P16. In each schema the letters are grouped in sectors proposed to the patient: in (A) and (B) “yellow”, “red”, “green”, “blue” and “white”, in (C) “1”, “2”, “3” and “4”, and in (D) “1”, “2”, “3”, “4” and “5”. The special characters represent in (A) and (B) “space” and “backspace”, and in (C) and (D) “space”, “backspace” and “delete word”.

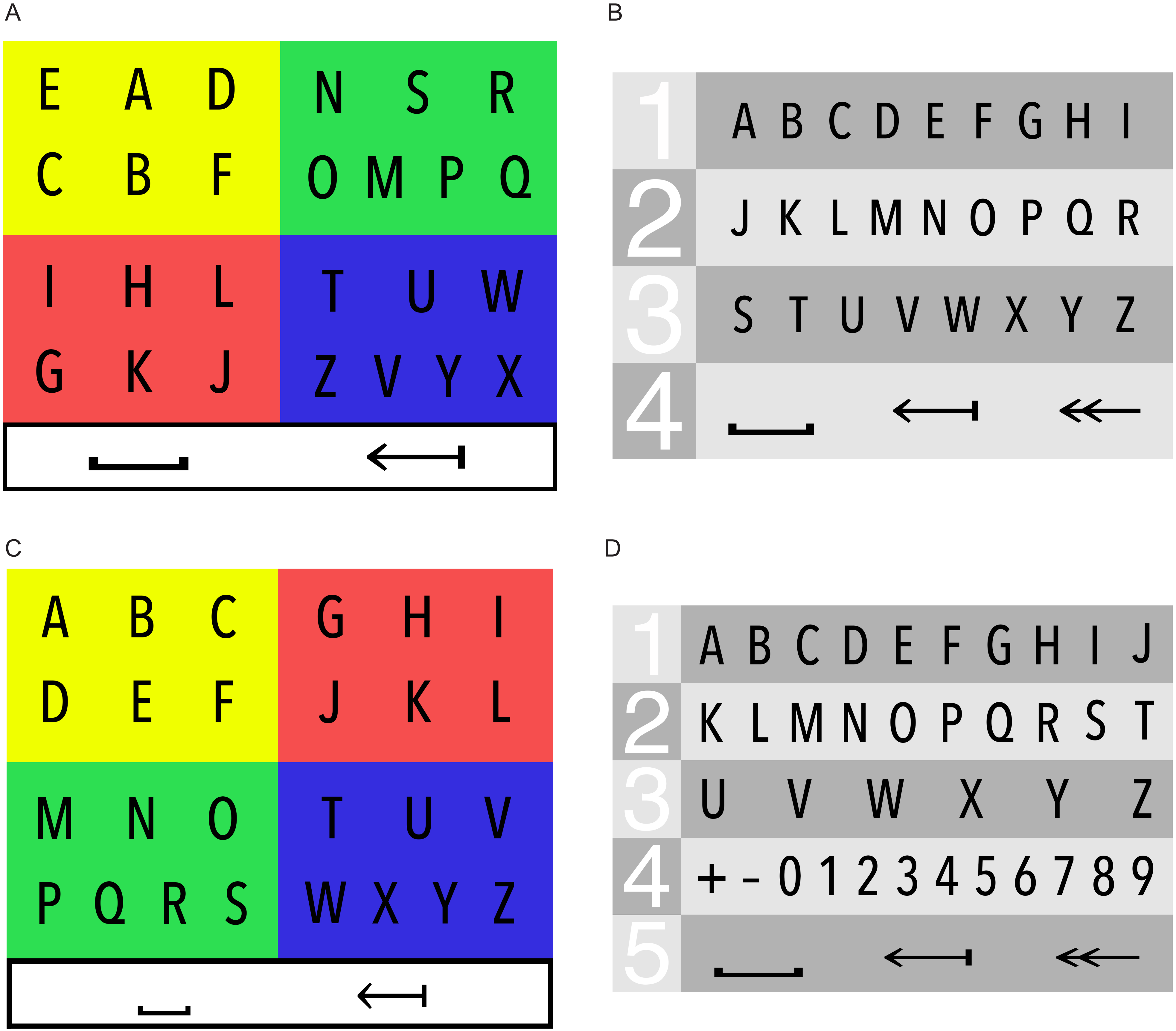
A
E A D
C B F
N S R
O M P Q
I H L
G K J
T U W
Z V Y X
B
1 A B C D E F G H I
2 J K L M N O P Q R
3 S T U V W X Y Z
4
C
A B C
D E F
G H I
J K L
M N O
P Q R S
T U V
W X Y Z
D
1 A B C D E F G H I J
2 K L M N O P Q R S T
3 U V W X Y Z
4 + - 0 1 2 3 4 5 6 7 8 9
5

Auditory Electrooculogram-based Communication System for ALS Patients in Transition from Locked-in to Complete Locked-in State

Alessandro Tonin[1+], Andres Jaramillo-Gonzalez[1+], Aygul Rana[1], Majid Khalili Ardali[1],

Niels Birbaumer[1,2], Ujwal Chaudhary[1,2*]

[1]Institute of Medical Psychology and Behavioral Neurobiology, University of Tübingen, Germany

[2]Wyss-Center for Bio- and Neuro-Engineering, Geneva, Switzerland

+These authors contributed equally

* Corresponding author: chaudharyujwal@gmail.com

Supplementary Figure S2:

Flow chart of the letter selection algorithm.

Circles indicate questions presented by the computer, diamonds decisions done by the algorithm, and squares internal process of the algorithm.

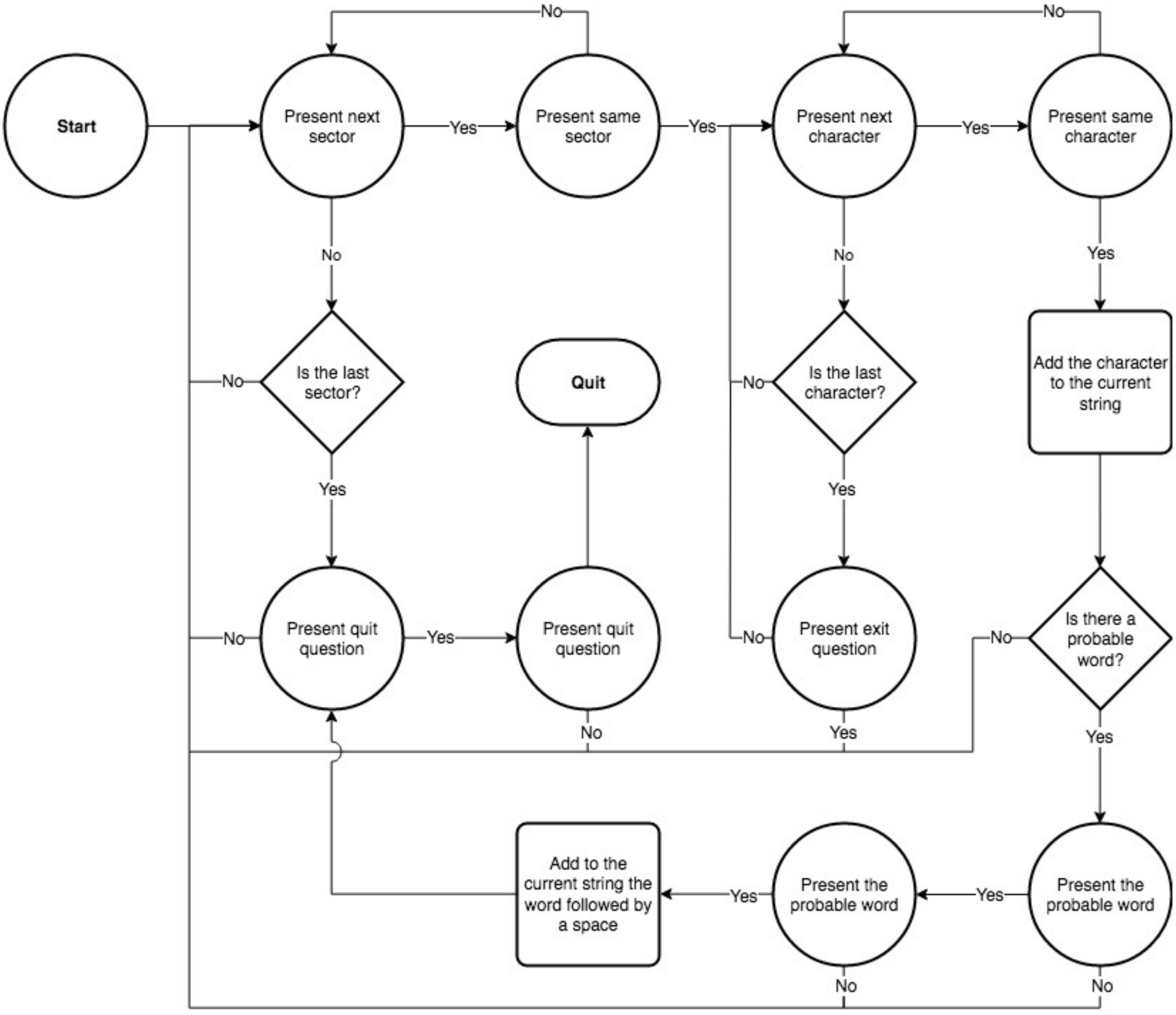
Start
Present next sector
Yes
Present same sector
No
Yes
Present next character
Yes
Present same character
No
No
Is the last sector?
No
Yes
Quit
No
Is the last character?
No
Yes
Yes
Add the character to the current string
Present quit question
No
Yes
Present quit question
No
Present exit question
No
Yes
Is there a probable word?
No
Yes
Add to the current string the word followed by a space
Yes
Present the probable word
Yes
Present the probable word
No
No

Auditory Electrooculogram-based Communication System for ALS Patients in Transition from Locked-in to Complete Locked-in State

Alessandro Tonin[1+], Andres Jaramillo-Gonzalez[1+], Aygul Rana[1], Majid Khalili Ardali[1],

Niels Birbaumer[1,2], Ujwal Chaudhary[1,2*]

[1]Institute of Medical Psychology and Behavioral Neurobiology, University of Tübingen, Germany

[2]Wyss-Center for Bio- and Neuro-Engineering, Geneva, Switzerland

+These authors contributed equally

* Corresponding author: chaudharyujwal@gmail.com

Supplementary Table S1:

Sequence of the training, feedback, and spelling sessions for P11.

Supplementary Table S1:
Sequence of the training, feedback and spelling sessions for P11

Visit	Day	Session	Date	Type of Session	Sequence of Sessions used for building the Prediction Model and Model Accuracy (%)	Prediction Accuracy (%)	Feature used for building the Online Model	Electrodes Used for Building the Online Model	Time length (in seconds) of the analyzed Response
V01	D01	S01	24-Mar-18	Training (lost raw file)					5.00
	D01	S02	24-Mar-18	Training (lost raw file)					5.00
	D01	S03	24-Mar-18	Training					5.00
	D01	S04	24-Mar-18	Feedback	D01(S02+S03) = 85%	30.00%	Maximum and Minimum (Time Domain)	7 EEG + 3 EOG	5.00
	D01	S05	24-Mar-18	Feedback	D01(S02+S03+S04) = 85%	50.00%	Maximum and Minimum (Time Domain)	7 EEG + 3 EOG	5.00
	D01	S06	24-Mar-18	Feedback	D01(S02+S03+S04+S05) = 85%	95.00%	Maximum and Minimum (Time Domain)	7 EEG + 3 EOG	5.00
	D02	S01	25-Mar-18	Feedback	D01(S02+S03+S04+S05+S06) = 90%	100.00%	Maximum and Minimum (Time Domain)	7 EEG + 3 EOG	5.00
	D02	S02	25-Mar-18	Feedback	D01(S02+S03+S04+S05+S06) = 90%	100.00%	Maximum and Minimum (Time Domain)	7 EEG + 3 EOG	5.00
	D02	S03	25-Mar-18	*					5.00
	D03	S01	26-Mar-18	Feedback	D01(S02+S03+S04+S05+S06)+D02(S01+S02) = 91.25%	85% (no ROC)	Maximum and Minimum (Time Domain)	7 EEG + 3 EOG	5.00
	D03	S02	26-Mar-18	Feedback	D01(S02+S03+S04+S05+S06)+D02(S01+S02) = 91.25%	95.00%	Maximum and Minimum (Time Domain)	7 EEG + 3 EOG	5.00
	D03	S03	26-Mar-18	*					5.00
	D03	S04	26-Mar-18	*					5.00
V02	D01	S01	2-May-18	Training					5.00
	D01	S02	2-May-18	Training					5.00
	D01	S03	2-May-18	Feedback	D01(S01+S02) = 91.66%	90.00%	Maximum and Minimum (Time Domain)	7 EEG + 4 EOG	5.00
	D01	S04	2-May-18	*					5.00
	D01	S05	2-May-18	*					5.00
	D02	S01	3-May-18	Feedback	D01(S01+S02+S03) = 90%	35.00%	Maximum and Minimum (Time Domain)	7 EEG + 4 EOG	5.00
	D02	S02	3-May-18	Feedback		50.00%	Maximum and Minimum (Time Domain)		5.00
	D02	S03	3-May-18	Training					5.00
	D02	S04	3-May-18	Feedback	D02(S01+S02+S03) = 90%	100.00%	Maximum and Minimum (Time Domain)	7 EEG + 4 EOG	5.00
	D03	S01	4-May-18	Training				7 EEG + 4 EOG	5.00
	D03	S02	4-May-18	Training					5.00
	D03	S03	4-May-18	Feedback	02(S01+S02+S03)+D03(S01+S02) = 80%	80.00%	Maximum and Minimum (Time Domain)	7 EEG + 4 EOG	5.00
	D03	S04	4-May-18	*					5.00
	D03	S05	4-May-18	*					5.00
	D04	S01	5-May-18	Training					5.00
	D04	S02	5-May-18	Feedback	D02(S01+S02+S03)+D03(S01+S02+S03)+D04(S01) = 83.63%	90.00%	Maximum and Minimum (Time Domain)	7 EEG + 4 EOG	5.00
	D04	S03	5-May-18	*					5.00
	D04	S04	5-May-18	*					5.00
V04	D01	S01	21-Aug-18	Training (Motor Attempt)					5.00
	D01	S02	21-Aug-18	Training (Motor Attempt)					5.00
	D01	S03	21-Aug-18	Training (Motor Attempt)					5.00
	D01	S04	21-Aug-18	Training (Motor Attempt)					5.00
	D01	S05	21-Aug-18	Training (Motor Attempt)					5.00
	D01	S06	21-Aug-18	Training (Motor Attempt)					5.00
	D01	S07	21-Aug-18	Training (Motor Attempt)					5.00
	D01	S08	21-Aug-18	Training (Motor Attempt)					5.00
	D01	S09	21-Aug-18	Training (Motor Attempt)					5.00
	D02	S01	22-Aug-18	Training					5.00
	D02	S02	22-Aug-18	Training					5.00
	D02	S03	22-Aug-18	Training					5.00
	D02	S04	22-Aug-18	Feedback	D02(S01+S02+S03) = 85%	90.00%	Maximum and Minimum (Time Domain)	12 EEG + 2 EOG	5.00
	D02	S05	22-Aug-18	Training (Motor Attempt)					5.00
	D02	S06	22-Aug-18	Training (Motor Attempt)					5.00
	D02	S07	22-Aug-18	Training (Motor Attempt)					5.00
	D02	S08	22-Aug-18	Training (Motor Attempt)					5.00
	D02	S09	22-Aug-18	Training (Motor Attempt)					5.00
	D02	S10	22-Aug-18	Training (Motor Attempt)					5.00
	D03	S01	23-Aug-18	Training (Motor Attempt)					5.00
	D03	S02	23-Aug-18	Training (Motor Attempt)					5.00
	D03	S03	23-Aug-18	Training (Motor Attempt)					5.00
	D03	S04	23-Aug-18	Feedback (Motor Attempt)	D03(S01+S02+S03) = 70%	50.00%	Mean of the Power Spectrum	12 EEG + 4 EOG	5.00
	D03	S05	23-Aug-18	Feedback (Motor Attempt)	D03(S02+S03) = 75.5%	50.00%	Peak of the Power Spectrum	12 EEG + 4 EOG	5.00
	D03	S06	23-Aug-18	Training (Motor Attempt)					5.00
	D03	S07	23-Aug-18	Training (Motor Attempt)					5.00
	D04	S01	24-Aug-18	Training					5.00
	D04	S02	24-Aug-18	Feedback	D03(S02+S03)+d4(S01) = 90%	90.00%	Maximum and Minimum (Time Domain)	12 EEG + 4 EOG	5.00
	D04	S03	24-Aug-18	*					5.00
	D04	S04	24-Aug-18	*					5.00
	D04	S05	24-Aug-18	*					5.00
	D04	S06	24-Aug-18	*					3.00

**Supplementary Table S1:
Sequence of the training, feedback and spelling sessions for P11**

	D04	S07	24-Aug-18	*					3.00
	D04	S08	24-Aug-18	*					3.00
	D04	S09	24-Aug-18	*					3.00
	D04	S10	24-Aug-18	*					3.00
	D04	S11	24-Aug-18	*					3.00
	D04	S12	24-Aug-18	*					3.00
	D04	S13	24-Aug-18	*					3.00
	D04	S14	24-Aug-18	*					3.00
	D04	S15	24-Aug-18	*					3.00
	D01	S01	19-Sep-18	Training					3.00
	D01	S02	19-Sep-18	Training					3.00
	D01	S03	19-Sep-18	Training					3.00
	D01	S04	19-Sep-18	Feedback	D01(S01+S03) = 90%	55.00%	Maximum and Minimum (Time Domain)	12 EEG + 4 EOG	3.00
	D01	S05	19-Sep-18	Feedback	D01(S01+S03+S04) = 85%	50.00%	Maximum and Minimum (Time Domain)	12 EEG + 4 EOG	3.00
	D02	S01	20-Sep-18	Training					3.00
	D02	S02	20-Sep-18	Feedback	D01(S01+S03+S04)+D02(S01) = 86%	90.00%	Maximum and Minimum (Time Domain)	12 EEG + 4 EOG	3.00
	D02	S03	20-Sep-18	*					3.00
	D02	S04	20-Sep-18	*					3.00
	D02	S05	20-Sep-18	*					3.00
	D02	S06	20-Sep-18	*					3.00
	D02	S07	20-Sep-18	*					3.00
V05	D03	S01	21-Sep-18	Training					3.00
	D03	S02	21-Sep-18	Feedback	D01(S01+S03+S04)+D02(S01+S02)+D03(S01) = 84.28%	85.00%	Maximum and Minimum (Time Domain)	12 EEG + 4 EOG	3.00
	D03	S03	21-Sep-18	*					3.00
	D03	S04	21-Sep-18	*					3.00
	D03	S05	21-Sep-18	*					3.00
	D03	S06	21-Sep-18	*					3.00
	D03	S07	21-Sep-18	*					3.00
	D03	S08	21-Sep-18	*					3.00
	D04	S01	22-Sep-18	Training					3.00
	D04	S02	22-Sep-18	Feedback	D01(S01+S03+S04)+D02(S01+S02)+D03(S01+S02)+D04(S01) = 82.20%	85.00%	Maximum and Minimum (Time Domain)	12 EEG + 4 EOG	3.00
	D04	S03	22-Sep-18	*					3.00
	D04	S04	22-Sep-18	*					3.00
	D04	S05	22-Sep-18	*					3.00
	D01	S01	5-Nov-18	Training					3.00
	D01	S02	5-Nov-18	Training					3.00
	D01	S03	5-Nov-18	Feedback	D01(S01+S02) = 92.50%	15.00%	Maximum and Minimum (Time Domain)	12 EEG + 3 EOG	3.00
	D01	S04	5-Nov-18	Feedback	D01(S01+S02+S03) = 86.60%	90.00%	Maximum and Minimum (Time Domain)	12 EEG + 3 EOG	3.00
	D01	S05	5-Nov-18	Feedback	D01(S01+S02+S03+S04) = 91.20%	85.00%	Maximum and Minimum (Time Domain)	12 EEG + 3 EOG	3.00
	D01	S06	5-Nov-18	*					3.00
	D01	S07	5-Nov-18	Speller					3.00
	D02	S01	6-Nov-18	Training					3.00
	D02	S02	6-Nov-18	Training					3.00
	D02	S03	6-Nov-18	Feedback	D02(S01+S02) = 92.50%	80.00%	Maximum and Minimum (Time Domain)	12 EEG + 3 EOG	3.00
	D02	S04	6-Nov-18	Feedback	D02(S01+S02+S03) = 85%	85.00%	Maximum and Minimum (Time Domain)	12 EEG + 3 EOG	3.00
	D02	S05	6-Nov-18	*					3.00
	D02	S06	6-Nov-18	*					3.00
	D02	S07	6-Nov-18	Speller					3.00
V06	D02	S08	6-Nov-18	Speller					3.00
	D02	S09	6-Nov-18	Speller					3.00
	D02	S10	6-Nov-18	Speller					3.00
	D03	S01	7-Nov-18	Training					3.00
	D03	S02	7-Nov-18	Feedback	D02(S01+S02+S03+S04) = Unknown result	50.00%	Maximum and Minimum (Time Domain)	12 EEG + 3 EOG	3.00
	D03	S03	7-Nov-18	Feedback	D02(S01+S02+S03+S04)+D03(S02) = 85%	75.00%	Maximum and Minimum (Time Domain)	12 EEG + 3 EOG	3.00
	D03	S04	7-Nov-18	Feedback	D02(S01+S02+S03+S04)+D03(S02+S03) = 82.50%	75.00%	Maximum and Minimum (Time Domain)	12 EEG + 3 EOG	3.00
	D03	S05	7-Nov-18	Feedback	D03(S02+S03+S04) = 75%	80.00%	Maximum and Minimum (Time Domain)	12 EEG + 3 EOG	3.00
	D03	S06	7-Nov-18	Feedback	D03(S02+S03+S04+S05) = 83.75%	95.00%	Maximum and Minimum (Time Domain)	12 EEG + 3 EOG	3.00
	D03	S07	7-Nov-18	*					3.00
	D03	S08	7-Nov-18	*					3.00
	D03	S09	7-Nov-18	Speller					3.00
	D03	S10	7-Nov-18	Speller					3.00
	D03	S11	7-Nov-18	Speller					3.00
	D02	S01	12-Dec-18	Training				11 EEG + 4 EOG	3.00
	D02	S02	12-Dec-18	Training				11 EEG + 4 EOG	3.00
	D02	S03	12-Dec-18	Feedback	D02(S02+S03) = 85%	85.00%	Maximum and Minimum (Time Domain)	11 EEG + 4 EOG	3.00
	D02	S04	12-Dec-18	Feedback	D02(S02+S03+S04) = 88.33%	95.00%	Maximum and Minimum (Time Domain)	11 EEG + 4 EOG	3.00
	D02	S05	12-Dec-18	*					3.00

Supplementary Table S1:
Sequence of the training, feedback and spelling sessions for P11

V07	D02	S06	12-Dec-18	Speller					3.00
	D02	S07	12-Dec-18	Speller					3.00
	D02	S08	12-Dec-18	Speller					3.00
	D02	S09	12-Dec-18	Speller					3.00
	D02	S10	12-Dec-18	Speller					3.00
	D02	S11	12-Dec-18	Speller					3.00
	D03	S01	13-Dec-18	Training					3.00
	D03	S02	13-Dec-18	Training					3.00
	D03	S03	13-Dec-18	Feedback	D03(S02+S03) = 77.50%	50.00%	Mean of the Power Spectrum	11 EEG + 4 EOG	3.00
	D03	S04	13-Dec-18	Training					5.00
	D03	S05	13-Dec-18	Feedback	D02(S02+S03+S04+S05)+D03(S05) = 88%	80.00%	Maximum and Minimum (Time Domain)	11 EEG + 4 EOG	5.00
	D03	S06	13-Dec-18	*					5.00
	D03	S07	13-Dec-18	*					5.00
	D03	S08	13-Dec-18	*					5.00
	D03	S09	13-Dec-18	Feedback	D03(S05+S06) = 95%	55.00%	Maximum and Minimum (Time Domain)	11 EEG + 4 EOG	5.00
V08	D01	S01	23-Jan-19	Training					5.00
	D01	S02	23-Jan-19	Training					5.00
	D01	S03	23-Jan-19	Training					10.00
	D01	S04	23-Jan-19	Training					10.00
	D01	S05	23-Jan-19	Feedback	D01(S03+S04) = 85%	85.00%	Maximum and Minimum (Time Domain)	12 EEG + 4 EOG	10.00
	D01	S06	23-Jan-19	Speller					10.00
	D01	S07	23-Jan-19	Speller					10.00
	D02	S01	24-Jan-19	Training					10.00
	D02	S02	24-Jan-19	Feedback	D01(S03+S04+S05)+D02(S02) = 82.50%	80.00%	Maximum and Minimum (Time Domain)	12 EEG + 4 EOG	10.00
	D02	S03	24-Jan-19	Speller					10.00
	D02	S04	24-Jan-19	Speller					10.00
	D02	S05	24-Jan-19	Speller					10.00
	D03	S01	25-Jan-19	Training					10.00
	D03	S02	25-Jan-19	Feedback	D01(S03+S04+S05)+D02(S03+S04) = 78%	85.00%	Maximum and Minimum (Time Domain)	12 EEG + 4 EOG	10.00
	D03	S03	25-Jan-19	*					10.00
	D03	S04	25-Jan-19	*					10.00
	D03	S05	25-Jan-19	Feedback	D01(S03+S04+S05)+D02(S03+S04) = 78%	60.00%	Maximum and Minimum (Time Domain)	12 EEG + 4 EOG	10.00
	D03	S06	25-Jan-19	Feedback (False Start)	D01(S03+S04+S05)+D02(S03+S04) = 78%	False Start	Maximum and Minimum (Time Domain)	12 EEG + 4 EOG	10.00
	D03	S07	25-Jan-19	Feedback	D01(S03+S04+S05)+D02(S03+S04) = 78%	85.00%	Maximum and Minimum (Time Domain)	12 EEG + 4 EOG	10.00
	D03	S08	25-Jan-19	Speller					10.00
	D03	S09	25-Jan-19	Speller					10.00
	D04	S01	26-Jan-19	Training					10.00
	D04	S02	26-Jan-19	Feedback	D01(S03+S04+S05)+D02(S03+S04)+D03(S02+S08)+D04(S01) = Unknown Result	65.00%	Maximum and Minimum (Time Domain)	12 EEG + 4 EOG	10.00
	D04	S03	26-Jan-19	Feedback	D01(S03+S04+S05)+D02(S03+S04)+D03(S02+S08)+D04(S02) = 78.75%	75.00%	Maximum and Minimum (Time Domain)	12 EEG + 4 EOG	10.00
	D04	S04	26-Jan-19	Feedback	D04(S02+S03) = 70%	55.00%	Mean of the Power Spectrum	12 EEG + 4 EOG	10.00
	D04	S05	26-Jan-19	Feedback	D04(S02+S03+S04) = 76.60%	75.00%	Maximum and Minimum (Time Domain)	12 EEG + 4 EOG	10.00
V09	D01	S01	14-Feb-19	Training					3.00
	D01	S02	14-Feb-19	Training					3.00
	D01	S03	14-Feb-19	Feedback	D01(S02+S03) = 75%	60.00%	Maximum and Minimum (Time Domain)	7 EEG + 4 EOG	3.00
	D01	S04	14-Feb-19	Feedback	D01(S02+S03+S04) = 76.70%	70.00%	Maximum and Minimum (Time Domain)	7 EEG + 4 EOG	3.00
	D01	S05	14-Feb-19	Feedback	D01(S02+S03+S04+S05) = 78.75%	60.00%	Maximum and Minimum (Time Domain)	7 EEG + 4 EOG	3.00
	D01	S06	14-Feb-19	Training					4.00
	D01	S07	14-Feb-19	Training					4.00
	D01	S08	14-Feb-19	Feedback	D01(S07+S08) = 85%	65.00%	Maximum and Minimum (Time Domain)		4.00
	D01	S09	14-Feb-19	Speller					4.00
	D02	S01	15-Feb-19	Training				7 EEG + 4 EOG	4.00
	D02	S02	15-Feb-19	Feedback	D01(S06+S07+S08)+D02(S01) = 78.75%.	55.00%	Maximum and Minimum (Time Domain)	7 EEG + 4 EOG	4.00
	D02	S03	15-Feb-19	Feedback	D02(S02+S03) = 85%	85.00%	Maximum and Minimum (Time Domain)	7 EEG + 4 EOG	4.00
	D02	S04	15-Feb-19	Speller					4.00
	D02	S05	15-Feb-19	Speller					4.00
V10	D01	S01	16-Mar-19	Training					5.00
	D01	S02	16-Mar-19	Training					5.00
	D01	S03	16-Mar-19	Training					5.00
	D01	S04	16-Mar-19	Training					5.00
	D01	S05	16-Mar-19	Training					8.00
	D01	S06	16-Mar-19	Training					8.00
	D01	S07	16-Mar-19	Feedback	D01(S04+S05+S06) = 65.00%	50.00%	Peak of the Power Spectrum	7 EEG + 4 EOG	8.00
	D01	S08	16-Mar-19	Feedback	D01(S04+S05+S06+S07) = 72.65%	50.00%	Maximum and Minimum (Time Domain)	7 EEG + 4 EOG	8.00
	D01	S09	16-Mar-19	Speller					8.00
	D01	S10	16-Mar-19	Speller					8.00
	D01	S11	16-Mar-19	Training					8.00
	D01	S12	16-Mar-19	Feedback	D01(S11) = 75.00%	50.00%	Maximum and Minimum (Time Domain)	7 EEG + 4 EOG	8.00

Supplementary Table S1: Sequence of the training, feedback and spelling sessions for P11

A detailed list of the sequence of visits (V), days (D), sessions (S), and the dates in which the session of the study were recorded from P11. The fifth column from left to right shows the type of session. Either Training, Feedback or Speller. Sessions indicated with (*) were recorded for a study outside the scope of this research or are defected recordings. In sessions indicated with (Motor Attempt), the experimenters asked the patient to produce Motor Attempt, instead of eye movement, to answer to the questions. The sixth column shows, only for the Feedback session, the sequence of sessions used for building the prediction model, e.g., "D01(S02+S03) = 85%" indicates that the model was build using the sessions S02 and S03 from day D01, meanwhile, 85% is the cross-validation accuracy achieved with this model. The seventh column shows the prediction accuracy achieved with the aforementioned model during the online experiments; those feedback sessions indicated as (no ROC) were not able to be included in Fig. 3, due to errors in the file of predicted categories. Columns eight and nine show the feature and number of electrodes used for building a model for the prediction. Column ten indicates the time length from which the feature was extracted.

Auditory Electrooculogram-based Communication System for ALS Patients in Transition from Locked-in to Complete Locked-in State

Alessandro Tonin[1+], Andres Jaramillo-Gonzalez[1+], Aygul Rana[1], Majid Khalili Ardali[1],

Niels Birbaumer[1,2], Ujwal Chaudhary[1,2*]

[1]Institute of Medical Psychology and Behavioral Neurobiology, University of Tübingen, Germany

[2]Wyss-Center for Bio- and Neuro-Engineering, Geneva, Switzerland

+These authors contributed equally

* Corresponding author: chaudharyujwal@gmail.com

Supplementary Table S2:

Sequence of the training, feedback, and spelling sessions for P13.

Supplementary Table S2:
Sequence of the training, feedback and spelling sessions for P13

Visit	Day	Session	Date	Type of Session	Sequence of Sessions used for building the Prediction Model and Model Accuracy (%)	Prediction Accuracy (%)	Feature used for building the Online Model	Electrodes Used for Building the Online Model	Time length (in seconds) of the analyzed Response
	D01	S01	20-Jun-18	Training					5.00
	D01	S02	20-Jun-18	Training					5.00
	D01	S03	20-Jun-18	Training					5.00
	D02	S01	21-Jun-18	Training					5.00
	D02	S02	21-Jun-18	Training					5.00
V01	D02	S03	21-Jun-18	Feedback	V01: D02(S01+S02) = 90%	100.00%	Maximum and Minimum (Time Domain)	7 EEG + 4 EOG	5.00
	D02	S04	21-Jun-18	Feedback	V01: D02(S01+S02+S03) = 90%	10.00%	Maximum and Minimum (Time Domain)	7 EEG + 4 EOG	5.00
	D03	S01	22-Jun-18	Training					5.00
	D03	S02	22-Jun-18	Feedback	V01: D02(S01+S02+S03)+D03(S01) = 91%	90.00%	Maximum and Minimum (Time Domain)	7 EEG + 4 EOG	5.00
	D03	S02	22-Jun-18	*					5.00
	D03	S03	22-Jun-18	*					5.00
	D01	S01	15-Oct-18	Training					5.00
	D01	S02	15-Oct-18	Training					5.00
	D01	S03	15-Oct-18	Training					5.00
	D01	S04	15-Oct-18	Feedback	V02: D01(S01+S02+S03) = 95%	95.00%	Maximum and Minimum (Time Domain)	7 EEG + EOGD, EOGR, EOGL	5.00
	D01	S05	15-Oct-18	Feedback	V02: D01(S04) = 93.75%	100.00%	Maximum and Minimum (Time Domain)	7 EEG + EOGD, EOGR, EOGL	5.00
	D01	S06	15-Oct-18	*					5.00
	D02	S01	16-Oct-18	Training					5.00
	D02	S02	16-Oct-18	Feedback	V02: D01(S01+S02+S03+S04)+D02(S01) = 93.33%	50.00%		7 EEG + EOGD, EOGR, EOGL	5.00
	D02	S03	16-Oct-18	Feedback	V02: D01(S01+S02+S03+S04)+D02(S01+S02) = 95.71%	95.00%	Maximum and Minimum (Time Domain)	7 EEG + EOGD, EOGR, EOGL	5.00
V02	D02	S04	16-Oct-18	*					5.00
	D02	S05	16-Oct-18	*					5.00
	D02	S06	16-Oct-18	*					5.00
	D03	S01	17-Oct-18	Training					5.00
	D03	S02	17-Oct-18	Feedback	V02: D01(S01+S02+S03+S04)+D02(S01+S02)+D03(S01) = Unknown Result	100.00%	Maximum and Minimum (Time Domain)	7 EEG + EOGD, EOGR, EOGL	5.00
	D03	S03	17-Oct-18	Speller					5.00
	D03	S04	17-Oct-18	Speller					5.00
	D03	S05	17-Oct-18	Speller					5.00
	D03	S06	17-Oct-18	Speller					5.00
	D03	S07	17-Oct-18	Speller					5.00
	D01	-	19-Feb-19	*					3.00
	D01	S01	19-Feb-19	Training					3.00
	D01	S02	19-Feb-19	Training					3.00
	D01	S03	19-Feb-19	Training					3.00
	D01	S04	19-Feb-19	Feedback	V03: D01(S01+S02+S03) = 100%	0.00%	Maximum and Minimum (Time Domain)	7 EEG + 4 EOG	3.00
	D01	S05	19-Feb-19	Feedback	V03: D01(S01+S02+S03+S04) = 100%	50.00%	Maximum and Minimum (Time Domain)	7 EEG + 4 EOG	3.00
	D02	S01	20-Feb-19	Training		-			5.00
	D02	S02	20-Feb-19	Training		-			5.00
	D02	S03	20-Feb-19	Feedback	V03: D02(S01+S02) = 100%	85.00%	Maximum and Minimum (Time Domain)	4 EOG	5.00
V03	D02	S04	20-Feb-19	Feedback	V03: D02(S01+S02+S03) = 90%	80.00%	Maximum and Minimum (Time Domain)	4 EOG	5.00
	D02	S05	20-Feb-19	Speller					5.00
	D02	S06	20-Feb-19	Speller					5.00
	D02	S07	20-Feb-19	Speller					5.00
	D02	S08	20-Feb-19	Speller					5.00
	D03	S01	21-Feb-19	Feedback	V03: D02(S01+S02+S03+S04) = 82%	90.00%	Maximum and Minimum (Time Domain)	4 EOG	5.00
	D03	S02	21-Feb-19	Speller					5.00
	D04	S01	22-Feb-19	Feedback	V03: D02(S01+S02+S03+S04)+D03(S01) = 84%	90.00%	Maximum and Minimum (Time Domain)	4 EOG	5.00
	D04	S02	22-Feb-19	Speller					5.00
	D04	S03	22-Feb-19	Speller					5.00
	D01	S01	28-May-19	Training					4.00
	D01	S02	28-May-19	Training					4.00
	D01	S03	28-May-19	Feedback	V04: D01(S01+S02) = 95%	5.00%	Maximum and Minimum (Time Domain)	4 EOG	4.00
	D01	S04	28-May-19	Feedback	V04: D01(S01+S02+S03) = 95%.	10.00%	Maximum and Minimum (Time Domain)	4 EOG	4.00
	D01	S05	28-May-19	Feedback	V04: D01(S01+S02+S03+S04) = 90%	15.00%	Maximum and Minimum (Time Domain)	4 EOG	4.00
	D02	S01	29-May-19	Training					4.00
	D02	S02	29-May-19	Training					4.00
	D02	S03	29-May-19	Training					4.00
	D02	S04	29-May-19	Training					4.00
	D02	S05	29-May-19	Feedback	V04: D02(S01+S04) = 90%	50.00%	Maximum and Minimum (Time Domain)	4 EOG	4.00
	D02	S06	29-May-19	Feedback	V04: D02(S01+S03) = 83%	50.00%	Maximum and Minimum (Time Domain)	4 EOG	4.00
	D02	S07	29-May-19	Feedback	V04: D02(S01+S02+S04) = 86.66%	75.00%	Maximum and Minimum (Time Domain)	4 EOG	4.00
	D02	S08	29-May-19	Speller					4.00
V04	D02	S09	29-May-19	Speller					4.00

Supplementary Table S2:
Sequence of the training, feedback and spelling sessions for P13

V04	D03	S01	30-May-19	Training					4.00
	D03	S02	30-May-19	Training					4.00
	D03	S03	30-May-19	Training					4.00
	D03	S04	30-May-19	Feedback	V04: D02(S01+S02+S03) = 92.5%	95.00%	Maximum and Minimum (Time Domain)	7 EEG + 4 EOG	4.00
	D03	S05	30-May-19	Speller					4.00
	D03	S06	30-May-19	Speller					4.00
	D03	S07	30-May-19	Speller					4.00
	D03	S08	30-May-19	Speller					4.00
	D04	S01	31-May-19	Training					4.00
	D04	S02	31-May-19	Training					4.00
	D04	S03	31-May-19	Training	V04: D03(S01+S04) = 90%	85.00%	Maximum and Minimum (Time Domain)	7 EEG + 4 EOG	4.00
	D04	S04	31-May-19	Speller					4.00
	D04	S05	31-May-19	Speller					4.00
	D04	S06	31-May-19	Speller					4.00

Supplementary Table S2: Sequence of the training, feedback and spelling sessions for P13

Detailed list of the sequence of visits (V), days (D), sessions (S), and the dates in which the session of the study were recorded from P13. The fifth column from left to right shows the type of session. Either Training, Feedback or Speller. Sessions indicated with (*) were recorded for a study outside the scope of this research or are defected recordings. The sixth column shows, only for the Feedback session, the sequence of sessions used for building the prediction model, e.g., “D01(S02+S03) = 85%“ indicates that the model was build using the sessions S02 and S03 from day D01, meanwhile, 85% is the cross-validation accuracy achieved with this model. The seventh column shows the prediction accuracy achieved with the aforementioned model, during the online experiments. Columns eight and nine show the feature and number of electrodes used for building a model for the prediction. Column ten indicates the time length from which the feature was extracted.

Auditory Electrooculogram-based Communication System for ALS Patients in Transition from Locked-in to Complete Locked-in State

Alessandro Tonin[1+], Andres Jaramillo-Gonzalez[1+], Aygul Rana[1], Majid Khalili Ardali[1],

Niels Birbaumer[1,2], Ujwal Chaudhary[1,2*]

[1]Institute of Medical Psychology and Behavioral Neurobiology, University of Tübingen, Germany

[2]Wyss-Center for Bio- and Neuro-Engineering, Geneva, Switzerland

+These authors contributed equally

* Corresponding author: chaudharyujwal@gmail.com

Supplementary Table S3:

Sequence of the training, feedback, and spelling sessions for P15.

Supplementary Table S3:
Sequence of the training, feedback and spelling sessions for P15

Visit	Day	Session	Date	Type of Session	Sequence of Sessions used for building the Prediction Model and Model Accuracy (%)	Prediction Accuracy (%)	Feature used for building the Online Model	Electrodes Used for Building the Online Model	Time length (in seconds) of the analyzed Response
V01	D01	S01	25-Feb-19	Training					3.00
	D01	S02	25-Feb-19	Training					3.00
	D01	S03	25-Feb-19	Training					3.00
	D01	S04	25-Feb-19	Feedback	V01: D01(S01+S02+S03) = 90%	50.00%	Maximum and Minimum (Time Domain)	7 EEG + 4 EOG	3.00
	D02	S01	26-Feb-19	Feedback	V01: D01(S01+S02+S03+S04) = 80%	30.00%	Maximum and Minimum (Time Domain)	7 EEG + 4 EOG	3.00
	D02	S02	26-Feb-19	Feedback	V01: D01(S01+S02+S03+S04)+D02(S01)= 85%	85.00%	Maximum and Minimum (Time Domain)	7 EEG + 4 EOG	3.00
	D03	S01	27-Feb-19	Training					3.00
	D03	S02	27-Feb-19	Feedback	V01: D03(S01) = 90%	90.00%	Maximum and Minimum (Time Domain)	4 EOG	3.00
	D03	S03	27-Feb-19	Speller					3.00
	D03	S04	27-Feb-19	Speller					3.00
	D03	S05	27-Feb-19	Feedback	V01: D03(S01+S02) = 100%	90.00%	Range: Maximum minus Minimum (Time Domain)		3.00
	D03	S06	27-Feb-19	Speller					3.00
	D03	S07	27-Feb-19	Speller					3.00
	D04	S01	28-Feb-19	Training					3.00
	D04	S02	28-Feb-19	Feedback	V01: D01(S01+S02+S03+S04)+D02(S01+S02)+D03(S01+S02+S05)+D04(S01) = 95%	95.00%	Range: Maximum minus Minimum (Time Domain)	4 EOG	3.00
	D04	S03	28-Feb-19	*					3.00
	D04	S04	28-Feb-19	*					3.00
	D04	S05	28-Feb-19	Speller					3.00
	D04	S06	28-Feb-19	Speller					3.00
	D04	S07	28-Feb-19	Speller					3.00
	D04	S08	28-Feb-19	Speller					3.00
	D04	S09	28-Feb-19	Speller					3.00
V02	D01	S01	25-Jun-19	Training					3.00
	D01	S02	25-Jun-19	Training					3.00
	D01	S03	25-Jun-19	Feedback	V02: D01(S01+S02) = 87.50%	25.00%	Range: Maximum minus Minimum (Time Domain)	4 EOG	3.00
	D01	S04	25-Jun-19	Feedback	V02: D01(S01+S02+S03) = 86.66%	15.00%	Range: Maximum minus Minimum (Time Domain)	4 EOG	3.00
	D01	S05	25-Jun-19	Feedback	V02: D01(S01+S02+S03) = 88.75%	95.00%	Range: Maximum minus Minimum (Time Domain)	4 EOG	3.00
	D01	S06	25-Jun-19	Speller					3.00
	D01	S07	25-Jun-19	Speller					3.00
	D02	S01	26-Jun-19	Feedback	V02: D01(S01+S02+S03) = 88.75%	95.00%	Range: Maximum minus Minimum (Time Domain)	4 EOG	3.00
	D02	S02	26-Jun-19	Speller					3.00
	D02	S03	26-Jun-19	Speller					3.00
	D02	S04	26-Jun-19	Speller					3.00
	D03	S01	27-Jun-19	Feedback	V02: D01(S01+S02+S03) = 88.75%	50% (no ROC)	Range: Maximum minus Minimum (Time Domain)	4 EOG	3.00
	D03	S02	27-Jun-19	Feedback	V02: D01(S01+S02+S03+S04) = 85%	70.00%	Range: Maximum minus Minimum (Time Domain)	4 EOG	3.00
	D03	S03	27-Jun-19	Feedback	V02: D01(S01+S02+S03+S04)+D03(S02) = 86%	50.00%	Range: Maximum minus Minimum (Time Domain)	4 EOG	3.00
	D03	S04	27-Jun-19	Feedback	V02: D03(S02+S03) = 92.50%	5.00%	Range: Maximum minus Minimum (Time Domain)	4 EOG	3.00
	D03	S05	27-Jun-19	Feedback	V02: D03(S02+S03+S04) = 93.33%	90.00%	Range: Maximum minus Minimum (Time Domain)	4 EOG	3.00
	D03	S06	27-Jun-19	Feedback	V02: D03(S02+S03+S04) = 93.75%	95.00%	Range: Maximum minus Minimum (Time Domain)	4 EOG	3.00
	D03	S07	27-Jun-19	Speller					3.00
	D03	S08	27-Jun-19	Speller					3.00
	D03	S09	27-Jun-19	Speller					3.00
	D03	S10	27-Jun-19	*					3.00
	D03	S11	27-Jun-19	*					3.00
	D03	S12	27-Jun-19	*					3.00
	D03	S13	27-Jun-19	*					3.00
	D03	S14	27-Jun-19	Speller					3.00

Supplementary Table S3: Sequence of the training, feedback and spelling sessions for P15

Detailed list of the sequence of visits (V), days (D), sessions (S), and the dates in which the session of the study were recorded from P15. The fifth column from left to right shows the type of session. Either Training, Feedback or Speller. Sessions indicated with (*) were recorded for a study outside the scope of this research or are defected recordings. The sixth column shows, only for the Feedback session, the sequence of sessions used for building the prediction model, e.g., “D01(S02+S03) = 85%“ indicates that the model was build using the sessions S02 and S03 from day D01, meanwhile, 85% is the cross-validation accuracy achieved with this model. The seventh column shows the prediction accuracy achieved with the aforementioned model during the online experiments; those feedback sessions indicated as (no ROC) were not able to be included in Fig. 3, due to errors in the file of predicted categories. Columns eight and nine show the feature and number of electrodes used for building a model for the prediction. Column ten indicates the time length from which the feature was extracted.

Auditory Electrooculogram-based Communication System for ALS Patients in Transition from Locked-in to Complete Locked-in State

Alessandro Tonin[1+], Andres Jaramillo-Gonzalez[1+], Aygul Rana[1], Majid Khalili Ardali[1],

Niels Birbaumer[1,2], Ujwal Chaudhary[1,2*]

[1]Institute of Medical Psychology and Behavioral Neurobiology, University of Tübingen, Germany

[2]Wyss-Center for Bio- and Neuro-Engineering, Geneva, Switzerland

+These authors contributed equally

* Corresponding author: chaudharyujwal@gmail.com

Supplementary Table S4:

Sequence of the training, feedback, and spelling sessions for P16.

Supplementary Table S4:
Sequence of the training, feedback and spelling sessions for P16

Visit	Day	Session	Date	Type of Session	Sequence of Sessions used for building the Prediction Model and Model Accuracy (%)	Prediction Accuracy (%)	Feature used for building the Online Model	Electrodes Used for Building the Online Model	Time length (in seconds) of the analyzed Response
V01	D01	S01	4-Mar-19	Training					3.00
	D01	S02	4-Mar-19	Training					3.00
	D01	S03	4-Mar-19	Training					3.00
	D01	S04	4-Mar-19	Training					3.00
	D01	S05	4-Mar-19	Feedback	V01: D01(S01+S02+S03+S04) = 97.5%	100.00%	Range: Maximum minus Minimum (Time Domain)	4 EOG	3.00
	D02	S01	5-Mar-19	*					3.00
	D02	S02	5-Mar-19	Feedback	V01: D01(S01+S02+S03+S04)+D01(S01) = 98.33%	50.00%	Range: Maximum minus Minimum (Time Domain)	4 EOG	3.00
	D02	S03	5-Mar-19	Feedback	V01: D02(S01+S02) = 92%	95.00%	Range: Maximum minus Minimum (Time Domain)	4 EOG	3.00
	D02	S04	5-Mar-19	Feedback	V01: D02(S01+S02+S03) = 100%	80.00%	Range: Maximum minus Minimum (Time Domain)	4 EOG	3.00
	D02	S05	5-Mar-19	*					3.00
	D02	S06	5-Mar-19	*					3.00
	D02	S07	5-Mar-19	Speller					3.00
	D02	S08	5-Mar-19	Speller					3.00
	D03	S01	6-Mar-19	Training					3.00
	D03	S02	6-Mar-19	Feedback	V01: D02(S01+S02+S03)+D03(S01) = 98%	50.00%	Range: Maximum minus Minimum (Time Domain)	4 EOG	3.00
	D03	S03	6-Mar-19	Feedback	V01: D03(S01+S02) = 100%	0.00%	Range: Maximum minus Minimum (Time Domain)	4 EOG	3.00
	D03	S04	6-Mar-19	Feedback	V01: D03(S01+S02+S03) = 100%	0.00%	Range: Maximum minus Minimum (Time Domain)	4 EOG	3.00
	D03	S05	6-Mar-19	*					3.00
	D03	S06	6-Mar-19	*					3.00
	D03	S07	6-Mar-19	Training					3.00
	D03	S08	6-Mar-19	Feedback	V01: D03(S07) = 95%	50.00%	Range: Maximum minus Minimum (Time Domain)	4 EOG	3.00
	D03	S09	6-Mar-19	Training					3.00
	D03	S10	6-Mar-19	Training					3.00
	D04	S01	7-Mar-19	Training					3.00
	D04	S02	7-Mar-19	Training					3.00
	D04	S03	7-Mar-19	Training					3.00
	D04	S04	7-Mar-19	Training					3.00
	D04	S05	7-Mar-19	Feedback	V01: D04(S01+S02+S03+S04) = 98.75%,	95.00%	Range: Maximum minus Minimum (Time Domain)	4 EOG	3.00
	D04	S06	7-Mar-19	Feedback	V01: D04(S01+S02+S03+S04+S05) = 99%,	90.00%	Range: Maximum minus Minimum (Time Domain)	4 EOG	3.00
	D04	S07	7-Mar-19	Speller					3.00
	D04	S08	7-Mar-19	*					3.00
	D05	S01	8-Mar-19	Training					3.00
	D05	S02	8-Mar-19	Training					3.00
	D05	S03	8-Mar-19	Training					3.00
	D05	S04	8-Mar-19	Feedback	V01: D05(S01+S02+S03) = 98.33%	90.00%	Range: Maximum minus Minimum (Time Domain)	4 EOG	3.00
	D05	S05	8-Mar-19	Speller					3.00
	D05	S06	8-Mar-19	Speller					3.00
V02	D01	S01	20-May-19	Training					4.00
	D01	S02	20-May-19	Training					4.00
	D01	S03	20-May-19	Training					4.00
	D01	S04	20-May-19	Training					4.00
	D01	S05	20-May-19	Training					4.00
	D01	S06	20-May-19	Training					4.00
	D01	S07	20-May-19	Training					4.00
	D02	S01	21-May-19	Training					4.00
	D02	S02	21-May-19	Training					4.00
	D02	S03	21-May-19	Feedback	V02: D02(S01+S02) = 95%	80.00%	Range: Maximum minus Minimum (Time Domain)	7 EEF, 4 EOG	4.00
	D02	S04	21-May-19	Feedback	V02: D02(S01+S02+S03) = 96.66%	45.00%	Range: Maximum minus Minimum (Time Domain)	7 EEF, 4 EOG	4.00
	D02	S05	21-May-19	Feedback	V02: D02(S01+S02) = 97.50%	100.00%	Range: Maximum minus Minimum (Time Domain)	7 EEF, 4 EOG	4.00
	D02	S06	21-May-19	Speller					4.00
	D03	S01	22-May-19	*					3.00
	D03	S02	22-May-19	Training					3.00
	D03	S03	22-May-19	Feedback	V02: D03(S01+S02) = 97.5%	65.00%	Range: Maximum minus Minimum (Time Domain)	7 EEF, 4 EOG	3.00
	D03	S04	22-May-19	Feedback	V02: D03(S01+02+03) = 100%	90.00%	Range: Maximum minus Minimum (Time Domain)	7 EEF, 4 EOG	3.00
	D03	S05	22-May-19	Speller					3.00
	D04	S01	23-May-19	Training					3.00
	D04	S02	23-May-19	Training					3.00
	D04	S03	23-May-19	Feedback	V02: D04(S01+S02) = 92.50%	50.00%	Range: Maximum minus Minimum (Time Domain)	7 EEF, 4 EOG	3.00
	D04	S04	23-May-19	Feedback	V02: D04(S01+S02) = 90%	50.00%	Range: Maximum minus Minimum (Time Domain)	7 EEF, 4 EOG	3.00
	D04	S05	23-May-19	Feedback	V02: D03(S01+S02+S03+S04) + D04(S01+S02) = 95%	70.00%	Range: Maximum minus Minimum (Time Domain)	7 EEF, 4 EOG	3.00
	D04	S06	23-May-19	Feedback	V02: D03(S01+S02+S03+S04) + D04(S01+S02+S03+S04+S05) = 93.88%	60.00%	Range: Maximum minus Minimum (Time Domain)	7 EEF, 4 EOG	3.00
	D04	S07	23-May-19	Feedback	V02: D04(S01+S02+S03+S04+S05+S06) = 95.83%	70.00%	Range: Maximum minus Minimum (Time Domain)	7 EEF, 4 EOG	3.00
	D04	S08	23-May-19	Feedback	V02: D04(S01+S02+S03+S04+S05+S06+S07) = 96,42%	85.00%	Range: Maximum minus Minimum (Time Domain)	7 EEF, 4 EOG	3.00

Supplementary Table S4:
Sequence of the training, feedback and spelling sessions for P16

	D04	S09	23-May-19	Speller					3.00

Supplementary Table S4: Sequence of the training, feedback and spelling sessions for P16

Detailed list of the sequence of visits (V), days (D), sessions (S), and the dates in which the session of the study were recorded from P16. The fifth column from left to right shows the type of session. Either Training, Feedback or Speller. Sessions indicated with (*) were recorded for a study outside the scope of this research or are defected recordings. The sixth column shows, only for the Feedback session, the sequence of sessions used for building the prediction model, e.g., “D01(S02+S03) = 85%“ indicates that the model was build using the sessions S02 and S03 from day D01, meanwhile, 85% is the cross-validation accuracy achieved with this model. The seventh column shows the prediction accuracy achieved with the aforementioned model, during the online experiments. Columns eight and nine show the feature and number of electrodes used for building a model for the prediction. Column ten indicates the time length from which the feature was extracted.

Auditory Electrooculogram-based Communication System for ALS Patients in Transition from Locked-in to Complete Locked-in State

Alessandro Tonin[1+], Andres Jaramillo-Gonzalez[1+], Aygul Rana[1], Majid Khalili Ardali[1],

Niels Birbaumer[1,2], Ujwal Chaudhary[1,2*]

[1]Institute of Medical Psychology and Behavioral Neurobiology, University of Tübingen, Germany

[2]Wyss-Center for Bio- and Neuro-Engineering, Geneva, Switzerland

+These authors contributed equally

* Corresponding author: chaudharyujwal@gmail.com

Supplementary Table S5:

Copy spelling sessions by the four patients.

Copy spelling sessions

Patient	Visit	Day	Session	Date	Target	Initial string	Selection	Speed (char/min)
P11	V06	D02	S09	06-Nov-18	ICH	-	ICH	0,85
	V06	D02	S10	06-Nov-18	BIN #####	-	BIN B#####	0,53
	V06	D03	S10	07-Nov-18	ICH BIN #####	-	ICH BIN #####	0,57
	V07	D02	S06	12-Dec-18	ICH BIN #####	-	NIE	0,57
	V07	D02	S07	12-Dec-18	ICH	-	ICH	0,96
	V07	D02	S08	12-Dec-18	BIN #####	ICH	DECNE	0,79
	V07	D02	S09	12-Dec-18	BIN #####	ICH	TN	0,34
	V07	D02	S10	12-Dec-18	BIN #####	ICH	BIN	0,42
	V07	D02	S11	12-Dec-18	#####	ICH BIN	#####	0,60
	V08	D01	S06	23-Jan-19	######	-	######	0,27
	V08	D02	S03	24-Jan-19	ICH	-	IET	0,33
	V08	D02	S04	24-Jan-19	ICH	-	D	0,28
	V09	D01	S09	14-Feb-19	####	-	####	0,11
	V10	D01	S09	16-Mar-19	JA	-	EEEEE	0,84
P13	V02	D03	S05	17-Oct-18	ICH	-	ICH	0,90
	V02	D03	S06	17-Oct-18	BIN ###	ICH	BIN ###	0,97
	V03	D02	S05	20-Feb-19	###	-	###	0,22
	V03	D02	S06	20-Feb-19	#####	-	#####	0,47
	V04	D02	S08	29-May-19	HALLO	-	AK	0,23
	V04	D02	S09	29-May-19	HALLO	-	HALKBLOO	0,54
	V04	D03	S05	30-May-19	###	-	###	0,29
	V04	D04	S04	31-May-19	###	-	###	0,15
P15	V01	D03	S04	27-Feb-19	ICH BIN #####	-	H	0,47
	V01	D04	S05	28-Feb-19	#####	-	#####	0,82
	V02	D01	S07	25-Jun-19	#####	-	H	0,11
	V02	D02	S02	26-Jun-19	#####	-	#####	0,44
	V02	D03	S07	27-Jun-19	#####	-	#####	0,22
P16	V01	D02	S07	05-Mar-19	######	-	CCAABA	1,15
	V01	D02	S08	05-Mar-19	HOX	-	GHONUAA	0,79
	V01	D04	S07	07-Mar-19	AUS	-	AUS	0,84
	V01	D05	S05	08-Mar-19	######	-	######	0,68

	V02	D02	S06	21-May-19	######	-	######	0,56

Supplementary Table S5: All copy spelling sessions by the four patients.
The columns indicate the patient, the session as visit, day and session number, the date, the target asked to select, the initial string already stored in the system (if any), the performed selection and the typing speed in characters per minute. Names of a patient or of a family member are replaced with #. Sessions have been excluded if an error in the code occurred, if the signal was noisy, if they terminated before 15 trials, if the patient did not select any letter, or if all the answers have been classified only as "yes" or only as "no": in total 6 sessions from P11, 2 sessions from P13, and 7 sessions from P15 were excluded.

Auditory Electrooculogram-based Communication System for ALS Patients in Transition from Locked-in to Complete Locked-in State

Alessandro Tonin[1+], Andres Jaramillo-Gonzalez[1+], Aygul Rana[1], Majid Khalili Ardali[1],

Niels Birbaumer[1,2], Ujwal Chaudhary[1,2*]

[1]Institute of Medical Psychology and Behavioral Neurobiology, University of Tübingen, Germany

[2]Wyss-Center for Bio- and Neuro-Engineering, Geneva, Switzerland

+These authors contributed equally

* Corresponding author: chaudharyujwal@gmail.com

Supplementary Table S6:

Free spelling sessions by the four patients.

Free spelling sessions

Patient	Visit	Day	Session	Date	Selection	Speed (char/min)
P11	V06	D02	S10*	06-Nov-18	[BIN B#####]ICH BRAUCHEN ANDERE STIMME	0,69
	V06	D03	S09	07-Nov-18	VSE	0,46
	V06	D03	S11	07-Nov-18	LICHT PO	0,48
	V07	D02	S11*	12-Dec-18	[ICH BIN #####]UND	1,01
	V08	D01	S07	23-Jan-19	UHR ZEIT	0,45
	V08	D02	S05	24-Jan-19	CAFE GRAAUES HERZ NNE KORNELIUS	0,33
	V09	D01	S09*	14-Feb-19	[####]PO I NEED SAV	0,24
	V09	D02	S04	15-Feb-19	ALX	0,20
	V09	D02	S05	15-Feb-19	OE J	0,22
P13	V02	D03	S07	17-Oct-18	DANKE	0,54
	V03	D02	S07	20-Feb-19	MUSSICHIMM	0,45
	V03	D02	S08	20-Feb-19	ZUVORFEATBACKMACHEN	0,41
	V03	D03	S02	21-Feb-19	ICH BIN FROHDASKINDERAUFWACHSENDARF	0,61
	V03	D04	S02	22-Feb-19	ICHMUSSP	0,63
	V03	D04	S03	22-Feb-19	FERNSEHEN KOMMT HEUTE U	1,02
	V04	D03	S06	30-May-19	ICH W	0,17
	V04	D03	S07	30-May-19	NAC	0,24
	V04	D03	S08	30-May-19	HHEBANZ	0,30
	V04	D04	S05	31-May-19	FENST	0,32
	V04	D04	S06	31-May-19	ICHFREUEAUFURLAUB	0,44
P15	V01	D04	S06	28-Feb-19	DDANKEUNE	0,56
	V02	D02	S02*	26-Jun-19	[#####]UND ##### LANZAROTE SKAT	0,81
	V02	D02	S03	26-Jun-19	PB	0,38
	V02	D02	S04	26-Jun-19	##### UND ####### HABEN HEUTE GEBURSTAG EURO FU	0,68
P16	V01	D05	S06	08-Mar-19	IAM HAPPY	0,63
	V02	D03	S05	22-May-19	MEINUNTERBEWUSST	0,52
	V02	D04	S09	23-May-19	SEIN HEILT ZELLEN	0,78

Supplementary Table S6: Free spelling sessions by the four patients.
The columns indicate the patient, the session as visit, day and session number, the date, the performed selection and the typing speed in characters per minute. Names of a patient or of a family member are replaced with #. In the sessions marked with * the patient started to spell immediately after the copy spelling with the same session name (Supplementary Table S5), the corresponding copy spelling selection is also reported inside brackets in the selection column. Sessions have been excluded if an error in the code occurred, if the signal was noisy, if they terminated before 15 trials, if the patient did not select any letter, or if all the answers have been classified only as “yes” or only as “no”: in total 3 sessions from P15 and 3 sessions from P16 were excluded.

Auditory Electrooculogram-based Communication System for ALS Patients in Transition from Locked-in to Complete Locked-in State

Alessandro Tonin[1+], Andres Jaramillo-Gonzalez[1+], Aygul Rana[1], Majid Khalili Ardali[1],

Niels Birbaumer[1,2], Ujwal Chaudhary[1,2*]

[1]Institute of Medical Psychology and Behavioral Neurobiology, University of Tübingen, Germany

[2]Wyss-Center for Bio- and Neuro-Engineering, Geneva, Switzerland

+These authors contributed equally

* Corresponding author: chaudharyujwal@gmail.com

Supplementary Table S7:

Session to Raw File-Recordings correspondence for all the patients.

Important Note:

The following tables include the correspondence between the Sessions Nomenclature and the available Raw Files.

Complexities of the *in-situ* recordings, adverse events, or the recording of sessions corresponding to other paradigms and studies, provoke that there is not an exact correspondence between the "Patient-Visit-Day-Session" Nomenclature, and the Raw Files. Therefore, the need for a table that allows to match the actual session and the available Raw Files.

In this tables there are more spelling sessions compared to the spelling sessions detailed in Supplementary Tables S5 and S6 of the paper. This is due to the fact that in those tables, sessions terminated completing 15 trials, or without selection of any letter have been excluded: in total 4 sessions from P11 and 6 sessions from P15 were excluded from Supplementary Table S5 and Supplementary Table S6.

Auditory Electrooculogram-based Communication System for ALS Patients in Transition from Locked-in to Complete Locked-in State

Supplementary Table S7: Session-Raw File Correspondence for P11

Patient	Visit	Day	Session	Date	Type of Session	Raw File Name (*.eeg)	Remark
	V01	D01	S01	24-Mar-18	Training	p11d1b1 (Lost)	Lost raw file
		D01	S02	24-Mar-18	Training	p11d1b2 (Lost)	Lost raw file
		D01	S03	24-Mar-18	Training	p11d1b3	
		D01	S04	24-Mar-18	Feedback	p11d1b4fb1	
		D01	S05	24-Mar-18	Feedback	p11d1b5fb2	
		D01	S06	24-Mar-18	Feedback	p11d1b6fb3Correct	
		D02	S01	25-Mar-18	Feedback	p11d2b1	
		D02	S02	25-Mar-18	Feedback	p11d2b2fb2	
		D02	S03	25-Mar-18	*		
		D03	S01	26-Mar-18	Feedback	p11d3b1fb1	Only 18 trials (w/o trigger 15), hardware error in initial 7 triggers
		D03	S02	26-Mar-18	Feedback	p11d3b1fb2	
		D03	S03	26-Mar-18	*		
		D03	S04	26-Mar-18	*		
	V02	D01	S01	2-May-18	Training	d1_0001	
		D01	S02	2-May-18	Training	d1_0002	
		D01	S03	2-May-18	Feedback	d1_0003	
		D01	S04	2-May-18	*		
		D01	S05	2-May-18	*		
		D02	S01	3-May-18	Feedback	d2_0001	
		D02	S02	3-May-18	Feedback	d2_0002	
		D02	S03	3-May-18	Training	d2_0003	
		D02	S04	3-May-18	Feedback	d2_0004	
		D03	S01	4-May-18	Training	d3_0001_ok	
		D03	S02	4-May-18	Training	d3_0002	
		D03	S03	4-May-18	Feedback	d3_0003	
		D03	S04	4-May-18	*		
		D03	S05	4-May-18	*		
		D04	S01	5-May-18	Training	d4_0001	Only 3 Trials
		D04	S02	5-May-18	Feedback	d4_0002	
		D04	S03	5-May-18	*		
		D04	S04	5-May-18	*		
	V04	D01	S01	21-Aug-18	Training (Motor Attempt)	p11_v04_d01_b01_trainingmotor	
		D01	S02	21-Aug-18	Training (Motor Attempt)	p11_v04_d01_b02_trainingmotor	
		D01	S03	21-Aug-18	Training (Motor Attempt)	p11_v04_d01_b03_trainingmotor	
		D01	S04	21-Aug-18	Training (Motor Attempt)	p11_v04_d01_b04_trainingmotor	
		D01	S05	21-Aug-18	Training (Motor Attempt)	p11_v04_d01_b05_trainingmotor	
		D01	S06	21-Aug-18	Training (Motor Attempt)	p11_v04_d01_b06_trainingmotor	
		D01	S07	21-Aug-18	Training (Motor Attempt)	p11_v04_d01_b07_trainingmotor	
		D01	S08	21-Aug-18	Training (Motor Attempt)	p11_v04_d01_b08_trainingmotor	
		D01	S09	21-Aug-18	Training (Motor Attempt)	p11_v04_d01_b09_trainingmotor	
		D02	S01	22-Aug-18	Training	p11_v04_d02_b1_training	
		D02	S02	22-Aug-18	Training	p11_v04_d02_b02_training	
		D02	S03	22-Aug-18	Training	p11_v04_d02_b03_training	
		D02	S04	22-Aug-18	Feedback	p11_v04_d02_b04_feedback	
		D02	S05	22-Aug-18	Training (Motor Attempt)	p11_v04_d02_b05_trainingmotor	
		D02	S06	22-Aug-18	Training (Motor Attempt)	p11_v04_d02_b06_trainingmotor	
		D02	S07	22-Aug-18	Training (Motor Attempt)	p11_v04_d02_b07_trainingmotor	
		D02	S08	22-Aug-18	Training (Motor Attempt)	p11_v04_d02_b08_trainingmotor	
		D02	S09	22-Aug-18	Training (Motor Attempt)	p11_v04_d02_b09_trainingmotor	
		D02	S10	22-Aug-18	Training (Motor Attempt)	p11_v04_d02_b10_trainingmotor	
		D03	S01	23-Aug-18	Training (Motor Attempt)	p11_v04_d03_b02_training	
		D03	S02	23-Aug-18	Training (Motor Attempt)	p11_v04_d03_b03_training	
		D03	S03	23-Aug-18	Training (Motor Attempt)	p11_v04_d03_b04_training	
		D03	S04	23-Aug-18	Feedback (Motor Attempt)	p11_v04_d03_b05_feedback	
		D03	S05	23-Aug-18	Feedback (Motor Attempt)	p11_v04_d03_b06_feedback	
		D03	S06	23-Aug-18	Training (Motor Attempt)	p11_v04_d03_b07_training	
		D03	S07	23-Aug-18	Training (Motor Attempt)	p11_v04_d03_b08_training	
		D04	S01	24-Aug-18	Training	p11_v04_d04_b01_training	
		D04	S02	24-Aug-18	Feedback	p11_v04_d04_b02_feedback	
		D04	S03	24-Aug-18	*		
		D04	S04	24-Aug-18	*		
		D04	S05	24-Aug-18	*		
		D04	S06	24-Aug-18	*		
		D04	S07	24-Aug-18	*		
		D04	S08	24-Aug-18	*		
		D04	S09	24-Aug-18	*		
		D04	S10	24-Aug-18	*		
		D04	S11	24-Aug-18	*		
		D04	S12	24-Aug-18	*		
		D04	S13	24-Aug-18	*		
		D04	S14	24-Aug-18	*		
		D04	S15	24-Aug-18	*		
		D01	S01	19-Sep-18	Training	p11_v05_d01_b02_training_01	
		D01	S02	19-Sep-18	Training	p11_v05_d01_b02_training_02	
		D01	S03	19-Sep-18	Training	p11_v05_d01_b04_training_03	
		D01	S04	19-Sep-18	Feedback	p11_v05_d01_b05_feedback_04	
		D01	S05	19-Sep-18	Feedback	p11_v05_d01_b06_feedback_05	
		D02	S01	20-Sep-18	Training	p11_v05_d02_b02_training01	
		D02	S02	20-Sep-18	Feedback	p11_v05_d02_b03_feedback02	
		D02	S03	20-Sep-18	*		
		D02	S04	20-Sep-18	*		
		D02	S05	20-Sep-18	*		
		D02	S06	20-Sep-18	*		
		D02	S07	20-Sep-18	*		

Supplementary Table S7: Session-Raw File Correspondence for P11

P11	V05	D03	S01	21-Sep-18	Training	p11_v05_d03_b02_training01	
		D03	S02	21-Sep-18	Feedback	p11_v05_d03_b03_feedback02	
		D03	S03	21-Sep-18	*		
		D03	S04	21-Sep-18	*		
		D03	S05	21-Sep-18	*		
		D03	S06	21-Sep-18	*		
		D03	S07	21-Sep-18	*		
		D03	S08	21-Sep-18	*		
		D04	S01	22-Sep-18	Training	p11_v05_d04_b02_training01	
		D04	S02	22-Sep-18	Feedback	p11_v05_d04_b03_feedback02	
		D04	S03	22-Sep-18	*		
		D04	S04	22-Sep-18	*		
		D04	S05	22-Sep-18	*		
	V06	D01	S01	5-Nov-18	Training	p11_v06_d01_b02_Training01	
		D01	S02	5-Nov-18	Training	p11_v06_d01_b03_Training02	
		D01	S03	5-Nov-18	Feedback	p11_v06_d01_b04_Feedback03	
		D01	S04	5-Nov-18	Feedback	p11_v06_d01_b05_Feedback04	
		D01	S05	5-Nov-18	Feedback	p11_v06_d01_b06_Feedback05	
		D01	S06	5-Nov-18	*		
		D01	S07	5-Nov-18	Speller	p11_v06_d01_b08_Speller07	w/o trigger 15 (bug while analyzing offline)
		D02	S01	6-Nov-18	Training	p11_v06_d02_b02_Training01	
		D02	S02	6-Nov-18	Training	p11_v06_d02_b03_Training02	
		D02	S03	6-Nov-18	Feedback	p11_v06_d02_b04_Feedback03	
		D02	S04	6-Nov-18	Feedback	p11_v06_d02_b05_Feedback04	
		D02	S05	6-Nov-18	*		
		D02	S06	6-Nov-18	*		
		D02	S07	6-Nov-18	Speller	p11_v06_d02_b08_Speller07	w/o trigger 15 (bug while analyzing offline)
		D02	S08	6-Nov-18	Speller	p11_v06_d02_b09_Speller08	w/o trigger 15
		D02	S09	6-Nov-18	Speller	p11_v06_d02_b10_Speller09	w/o trigger 15
		D02	S10	6-Nov-18	Speller	p11_v06_d02_b11_Speller10	w/o trigger 15
		D03	S01	7-Nov-18	Training	p11_v06_d03_b02_Training01	
		D03	S02	7-Nov-18	Feedback	p11_v06_d03_b03_Feedback02	
		D03	S03	7-Nov-18	Feedback	p11_v06_d03_b04_Feedback03	
		D03	S04	7-Nov-18	Feedback	p11_v06_d03_b05_Feedback04	
		D03	S05	7-Nov-18	Feedback	p11_v06_d03_b06_Feedback05	
		D03	S06	7-Nov-18	Feedback	p11_v06_d03_b07_Feedback06	
		D03	S07	7-Nov-18	*		
		D03	S08	7-Nov-18	*		
		D03	S09	7-Nov-18	Speller	p11_v06_d03_b10_Speller09	w/o trigger 15
		D03	S10	7-Nov-18	Speller	p11_v06_d03_b11_Speller10	
		D03	S11	7-Nov-18	Speller	p11_v06_d03_b12_Speller11	
	V07	D02	S01	12-Dec-18	Training	p11_v07_d02_b02_training01	
		D02	S02	12-Dec-18	Training	p11_v07_d02_b03_training02	
		D02	S03	12-Dec-18	Feedback	p11_v07_d02_b04_feedback03	
		D02	S04	12-Dec-18	Feedback	p11_v07_d02_b05_feedback04	
		D02	S05	12-Dec-18	*		
		D02	S06	12-Dec-18	Speller	p11_v07_d02_b07_speller06	
		D02	S07	12-Dec-18	Speller	p11_v07_d02_b08_speller07	
		D02	S08	12-Dec-18	Speller	p11_v07_d02_b09_speller08	w/o trigger 15
		D02	S09	12-Dec-18	Speller	p11_v07_d02_b10_speller09	w/o trigger 15
		D02	S10	12-Dec-18	Speller	p11_v07_d02_b11_speller10	
		D02	S11	12-Dec-18	Speller	p11_v07_d02_b12_speller11	
		D03	S01	13-Dec-18	Training	p11_v07_d03_b02_training01	
		D03	S02	13-Dec-18	Training	p11_v07_d03_b03_training02	
		D03	S03	13-Dec-18	Feedback	p11_v07_d03_b04_feedback03	
		D03	S04	13-Dec-18	Training	p11_v07_d03_b05_training04	
		D03	S05	13-Dec-18	Feedback	p11_v07_d03_b06_feedback05	
		D03	S06	13-Dec-18	*		
		D03	S07	13-Dec-18	*		
		D03	S08	13-Dec-18	*		
		D03	S09	13-Dec-18	Feedback	p11_v07_d03_b11_feedback09	
	V08	D01	S01	23-Jan-19	Training	p11_v08_d01_b02_Training01	
		D01	S02	23-Jan-19	Training	p11_v08_d01_b03_Training02	
		D01	S03	23-Jan-19	Training	p11_v08_d01_b04_Training03	
		D01	S04	23-Jan-19	Training	p11_v08_d01_b04_Training04	
		D01	S05	23-Jan-19	Feedback	p11_v08_d01_b06_Feedback05	
		D01	S06	23-Jan-19	Speller	p11_v08_d01_b07_Speller06	w/o trigger 15
		D01	S07	23-Jan-19	Speller	p11_v08_d01_b08_Speller07	
		D02	S01	24-Jan-19	Training	p11_v08_d02_b02_Training01	
		D02	S02	24-Jan-19	Feedback	p11_v08_d02_b03_Feedback02	
		D02	S03	24-Jan-19	Speller	p11_v08_d02_b04_Speller03	w/o trigger 15
		D02	S04	24-Jan-19	Speller	p11_v08_d02_b05_Speller04	w/o trigger 15
		D02	S05	24-Jan-19	Speller	p11_v08_d02_b06_Speller05	w/o trigger 15
		D03	S01	25-Jan-19	Training	p11_v08_d03_b02_training01	
		D03	S02	25-Jan-19	Feedback	p11_v08_d03_b03_feedback02	
		D03	S03	25-Jan-19	*		
		D03	S04	25-Jan-19	*		
		D03	S05	25-Jan-19	Feedback	p11_v08_d03_b06_feedback05	
		D03	S06	25-Jan-19	Feedback	p11_v08_d03_b07_feedback06	Only 3 Trials, session interrupted, (w/o trigger 15)
		D03	S07	25-Jan-19	Feedback	p11_v08_d03_b08_feedback07	
		D03	S08	25-Jan-19	Speller	p11_v08_d03_b09_speller08	Intruder Trigger "DCC" (bug while analyzing offline)
		D03	S09	25-Jan-19	Speller	p11_v08_d03_b10_speller09	Intruder Trigger "DCC" (bug while analyzing offline)

		D04	S01	26-Jan-19	Training	p11_v08_d04_b02_training	
		D04	S02	26-Jan-19	Feedback	p11_v08_d04_b03_feedback02	
		D04	S03	26-Jan-19	Feedback	p11_v08_d04_b04_feedback03	
		D04	S04	26-Jan-19	Feedback	p11_v08_d04_b05_feedback04	
		D04	S05	26-Jan-19	Feedback	p11_v08_d04_b06_feedback05	
		D01	S01	14-Feb-19	Training	p11_v09_d01_b02training01	
		D01	S02	14-Feb-19	Training	p11_v09_d01_b03training02	
		D01	S03	14-Feb-19	Feedback	p11_v09_d01_b04feedback03	
		D01	S04	14-Feb-19	Feedback	p11_v09_d01_b05feedback04	
		D01	S05	14-Feb-19	Feedback	p11_v09_d01_b06feedback06	
		D01	S06	14-Feb-19	Training	p11_v09_d01_b07training06	
	V09	D01	S07	14-Feb-19	Training	p11_v09_d01_b08training07	
		D01	S08	14-Feb-19	Feedback	p11_v09_d01_b09feedback08	
		D01	S09	14-Feb-19	Speller	p11_v09_d01_b10speller09	w/o trigger 15
		D02	S01	15-Feb-19	Training	p11_v09_d02_b02training01	
		D02	S02	15-Feb-19	Feedback	p11_v09_d02_b03feedback02	
		D02	S03	15-Feb-19	Feedback	p11_v09_d02_b03feedback03	
		D02	S04	15-Feb-19	Speller	p11_v09_d02_b05speller04	
		D02	S05	15-Feb-19	Speller	p11_v09_d02_b06speller05	w/o trigger 15
		D01	S01	16-Mar-19	Training	p11_v10_d01_b02_training01	
		D01	S02	16-Mar-19	Training	p11_v10_d01_b03_training02	
		D01	S03	16-Mar-19	Training	p11_v10_d01_b04_training03	
		D01	S04	16-Mar-19	Training	p11_v10_d01_b05_training04	
		D01	S05	16-Mar-19	Training	p11_v10_d01_b06_training05	
	V10	D01	S06	16-Mar-19	Training	p11_v10_d01_b07_training06	
		D01	S07	16-Mar-19	Feedback	p11_v10_d01_b08_feedback07	
		D01	S08	16-Mar-19	Feedback	p11_v10_d01_b09_feedback08	
		D01	S09	16-Mar-19	Speller	p11_v10_d01_b10_speller09	
		D01	S10	16-Mar-19	Speller	p11_v10_d01_b11_speller10	Intruder Triggers after the end of session
		D01	S11	16-Mar-19	Training	p11_v10_d01_b12_training11	
		D01	S12	16-Mar-19	Feedback	p11_v10_d01_b13_feedback12	

Supplementary Table S7:
Session-Raw File Correspondence for P11, Sequence of the training, feedback and spelling sessions.
A detailed list of the sequence of visits (V), days (D), sessions (S), the dates in which the session of the study was recorded from P11. The sixth column from left to right shows the type of session. Either Training, Feedback or Speller. Sessions indicated with (*) were recorded for a study outside the scope of this research or are defected recordings. In sessions indicated with (Motor Attempt), the experimenters asked the patient to produce Motor Attempt, instead of eye movement, to answer to the questions. Columns seven specifies the exact name of the raw file containing the recordings. Column eight gives some remarks of irregularities during the in situ recording of the raw file of the session.

Supplementary Table S7:
Session-Raw File Correspondence for P13

Patient	Visit	Day	Session	Date	Type of Session	Raw File Name (*.eeg)	Remark
P13	V01	D01	S01	20-Jun-18	Training	d1_0001	
		D01	S02	20-Jun-18	Training	d1_0002	
		D01	S03	20-Jun-18	Training	d1_0003	
		D02	S01	21-Jun-18	Training	d2_0001	
		D02	S02	21-Jun-18	Training	d2_0002	
		D02	S03	21-Jun-18	Feedback	d2_0003_fb1	
		D02	S04	21-Jun-18	Feedback	d2_0004_fb2	
		D03	S01	22-Jun-18	Training	d3_0001	
		D03	S02	22-Jun-18	Feedback	d3_0002_fb1	
		D03	S02	22-Jun-18	*		
		D03	S03	22-Jun-18	*		
	V02	D01	S01	15-Oct-18	Training	p13_v02_d01_b01_training01	
		D01	S02	15-Oct-18	Training	p13_v02_d01_b02_training02	
		D01	S03	15-Oct-18	Training	p13_v02_d01_b03_training03	
		D01	S04	15-Oct-18	Feedback	p13_v02_d01_b04_feedback01	
		D01	S05	15-Oct-18	Feedback	p13_v02_d01_b05_feedback02	
		D01	S06	15-Oct-18	*		
		D02	S01	16-Oct-18	Training	p13_v02_d02_b2_training1	
		D02	S02	16-Oct-18	Feedback	p13_v02_d02_b3_feedback1	
		D02	S03	16-Oct-18	Feedback	p13_v02_d02_b4_feedback2	
		D02	S04	16-Oct-18	*		
		D02	S05	16-Oct-18	*		
		D02	S06	16-Oct-18	*		
		D03	S01	17-Oct-18	Training	p13_v02_d03_b02_training01	
		D03	S02	17-Oct-18	Feedback	p13_v02_d03_b03_feedback01	
		D03	S03	17-Oct-18	Speller	p13_v02_d03_b04_speller01	20 complete trials (1 uncomplete) (w/o trigger 15) (bug offline)
		D03	S04	17-Oct-18	Speller	p13_v02_d03_b05_speller02	(bug while analyzing offline)
		D03	S05	17-Oct-18	Speller	p13_v02_d03_b06_speller03	
		D03	S06	17-Oct-18	Speller	p13_v02_d03_b07_speller04	
		D03	S07	17-Oct-18	Speller	p13_v02_d03_b08_speller05	68 complete trials (1 uncomplete) (w/o trigger 15)
	V03	D01	S01	19-Feb-19	Training	p13_v03_d01_b01_tr01	
		D01	S02	19-Feb-19	Training	p13_v03_d01_b02_fb01	
		D01	S03	19-Feb-19	Training	p13_v03_d01_b02_fb03	
		D01	S04	19-Feb-19	Feedback	p13_v03_d01_b02_fb04	
		D01	S05	19-Feb-19	Feedback	Contained in V03D01S04	Contained at he end of p13_v03_d01_b02_fb04. Only 10 trials
		D02	S01	20-Feb-19	Training	p13_v03_d02_b01_tr01	
		D02	S02	20-Feb-19	Training	p13_v03_d02_b02_tr02	
		D02	S03	20-Feb-19	Feedback	p13_v03_d01_fb01	
		D02	S04	20-Feb-19	Feedback	p13_v03_d02_b04_fb02	
		D02	S05	20-Feb-19	Speller	p13_v03_d02_b05_sp01	88 complete trials (1 uncomplete) (w/o trigger 15)
		D02	S06	20-Feb-19	Speller	p13_v03_d02_b06_sp02	
		D02	S07	20-Feb-19	Speller	p13_v03_d02_b07_sp03	
		D02	S08	20-Feb-19	Speller	p13_v03_d02_b08_sp04	
		D03	S01	21-Feb-19	Feedback	p13_v03_d03_b01_fb01	
		D03	S02	21-Feb-19	Speller	p13_v03_d03_b02_sp01	298 complete trials (1 uncomplete) (w/o trigger 15)
		D04	S01	22-Feb-19	Feedback	p13_v03_d04_b01_fb01	
		D04	S02	22-Feb-19	Speller	p13_v03_d04_b02_sp01	78 complete trials (1 uncomplete) (w/o trigger 15)
		D04	S03	22-Feb-19	Speller	p13_v03_d04_b03_sp02	
	V04	D01	S01	28-May-19	Training	p13_v04_d01_b0001	
		D01	S02	28-May-19	Training	p13_v04_d01_b0003	
		D01	S03	28-May-19	Feedback	p13_v04_d01_bf0003	7 initial triggers from a false start
		D01	S04	28-May-19	Feedback	p13_v04_d01_bf0004	
		D01	S05	28-May-19	Feedback	p13_v04_d01_bf005	
		D02	S01	29-May-19	Training	p13_v04_d02_b0001	
		D02	S02	29-May-19	Training	p13_v04_d02_b0002	
		D02	S03	29-May-19	Training	p13_v04_d02_btf0003	
		D02	S04	29-May-19	Training	p13_v04_d02_btf0004	
		D02	S05	29-May-19	Feedback	p13_v04_d02_bAF0005	
		D02	S06	29-May-19	Feedback	p13_v04_d02_baf0010	
		D02	S07	29-May-19	Feedback	p13_v04_d02_baf0011	
		D02	S08	29-May-19	Speller	p13_v04_d02_bSP0012	
		D02	S09	29-May-19	Speller	p13_v04_d02_bsp0013	
		D03	S01	30-May-19	Training	p13_v04_d03_b0001	
		D03	S02	30-May-19	Training	p13_v04_d03_b0002	
		D03	S03	30-May-19	Training	p13_v04_d03_btf0003	
		D03	S04	30-May-19	Feedback	p13_v04_d03_baf0004	
		D03	S05	30-May-19	Speller	p13_v04_d03_bSP0005	
		D03	S06	30-May-19	Speller	p13_v04_d03_bSp0006	
		D03	S07	30-May-19	Speller	p13_v04_d03_bSP0007	
		D03	S08	30-May-19	Speller	p13_v04_d03_bsp0008	
		D04	S01	31-May-19	Training	p13_v04_d04_b0001	
		D04	S02	31-May-19	Training	p13_v04_d04_b0002	
		D04	S03	31-May-19	Training	p13_v04_d04_btf0003	
		D04	S04	31-May-19	Speller	p13_v04_d04_bsp0004	
		D04	S05	31-May-19	Speller	p13_v04_d04_bsp0005	
		D04	S06	31-May-19	Speller	p13_v04_d04_bsp0006	179 complete trials (w/o trigger 15), parasite triggers at the end

Supplementary Table S7:
Session-Raw File Correspondence for P13, Sequence of the training, feedback and spelling sessions.
A detailed list of the sequence of visits (V), days (D), sessions (S), the dates in which the session of the study was recorded from P13. The sixth column from left to right shows the type of session. Either Training, Feedback or Speller. Sessions indicated with (*) were recorded for a study outside the scope of this research or are defected recordings. Columns seven specifies the exact name of the raw file containing the recordings. Column eight gives some remarks of irregularities during the in situ recording of the raw file of the session.

Supplementary Table S7:
Session-Raw File Correspondence for P15

Patient	Visit	Day	Session	Date	Type of Session	Raw File Name (*.eeg)	Remark
P15	V01	D01	S01	25-Feb-19	Training	p15_v01_d01_b01_training01	
		D01	S02	25-Feb-19	Training	p15_v01_d01_b02_feedback01	
		D01	S03	25-Feb-19	Training	p15_v01_d01_b03_feedback02	
		D01	S04	25-Feb-19	Feedback	p15_v01_d01_b04_feedback03	
		D02	S01	26-Feb-19	Feedback	p15_v01_d02_b01_feedback (Lost)	Lost raw file
		D02	S02	26-Feb-19	Feedback	p15_v01_d02_b02_feedback	(Feature modified w/baseline correction)
		D03	S01	27-Feb-19	Training	p15_v01_d03_b0001_training01	
		D03	S02	27-Feb-19	Feedback	p15_v01_d03_b02_feedback01	
		D03	S03	27-Feb-19	Speller	p15_v01_d03_b03_speller01	7 trials (w/o trigger 15) (only "yes")
		D03	S04	27-Feb-19	Speller	p15_v01_d03_b04_speller02_ok**	5 trials (w/o trigger 15)
		D03	S05	27-Feb-19	Feedback	p15_v01_d03_b05_feedback02**	Only ten questions
		D03	S06	27-Feb-19	Speller	p15_v01_d03_b06_speller03**	5 trials (w/o trigger 15)
		D03	S07	27-Feb-19	Speller	p15_v01_d03_b07_spelller04	16 trials (w/o trigger 15 (only "yes")
		D04	S01	28-Feb-19	Training	p15_v01_d04_b01_training	
		D04	S02	28-Feb-19	Feedback	p15_v01_d04_b02_feedback	
		D04	S03	28-Feb-19	*		
		D04	S04	28-Feb-19	*		
		D04	S05	28-Feb-19	Speller	p15_v01_d04_b05_speller01	
		D04	S06	28-Feb-19	Speller	p15_v01_d04_b06_speller02	112 trials (w/o trigger 15)
		D04	S07	28-Feb-19	Speller	p15_v01_d04_b07_speller03	9 trials (w/o trigger 15) (only "yes")
		D04	S08	28-Feb-19	Speller	p15_v01_d04_b08_speller04	4 trials (w/o trigger 15) (only "yes")
		D04	S09	28-Feb-19	Speller	p15_v01_d04_b09_speller05	8 trials (w/o trigger 15) (only "yes")
	V02	D01	S01	25-Jun-19	Training	p15_v02_d01_b01_training	
		D01	S02	25-Jun-19	Training	p15_v02_d01_b02_training02	
		D01	S03	25-Jun-19	Feedback	p15_v02_d01_b03_feedback	
		D01	S04	25-Jun-19	Feedback	p15_v02_d01_b04_feedback	
		D01	S05	25-Jun-19	Feedback	p15_v02_d01_b05_feedback	
		D01	S06	25-Jun-19	Speller	p15_v02_d01_b06_speller	28 trials (w/o trigger 15) (No selection made)
		D01	S07	25-Jun-19	Speller	p15_v02_d01_b07_speller	67 trials (w/o trigger 15)
		D02	S01	26-Jun-19	Feedback	p15_v02_d02_b02_training	
		D02	S02	26-Jun-19	Speller	p15_v02_d02_b03_speller	282 trials (w/o trigger 15)
		D02	S03	26-Jun-19	Speller	p15_v02_d02_b04_speller	
		D02	S04	26-Jun-19	Speller	p15_v02_d02_b05_speller	476 trials (w/o trigger 15)
		D03	S01	27-Jun-19	Feedback	p15_v02_d03_b02_feedback	Session stopped (w/o trigger 15)
		D03	S02	27-Jun-19	Feedback	p15_v02_d03_b03_feedback	
		D03	S03	27-Jun-19	Feedback	p15_v02_d03_b04_feedback	
		D03	S04	27-Jun-19	Feedback	p15_v02_d03_b05_feedback	
		D03	S05	27-Jun-19	Feedback	p15_v02_d03_b06_feedback	
		D03	S06	27-Jun-19	Feedback	p15_v02_d03_b07_feedback	
		D03	S07	27-Jun-19	Speller	p15_v02_d03_b08_speller	
		D03	S08	27-Jun-19	Speller	p15_v02_d03_b09_speller	2 trials (w/o trigger 15)
		D03	S09	28-Jun-19	Speller	p15_v02_d03_b10_speller	2 trials (w/o trigger 15)
		D03	S14	27-Jun-19	*		
		D03	S15	27-Jun-19	*		
		D03	S16	27-Jun-19	*		
		D03	S17	27-Jun-19	*		
		D03	S18	27-Jun-19	Speller	p15_v02_d03_b15_speller	48 trials (w/o trigger 15)

Supplementary Table S7:
Session-Raw File Correspondence for P15, Sequence of the training, feedback and spelling sessions.
A detailed list of the sequence of visits (V), days (D), sessions (S), the dates in which the session of the study was recorded from P15. The sixth column from left to right shows the type of session. Either Training, Feedback or Speller. Sessions indicated with (*) were recorded for a study outside the scope of this research or are defected recordings. Columns seven specifies the exact name of the raw file containing the recordings. Raw files indicated with (**) were segmented from an original continuous recording. Column eight gives some remarks of irregularities during the in situ recording of the raw file of the session.

Supplementary Table S7:
Session-Raw File Correspondence for P16

Patient	Visit	Day	Session	Date	Type of Session	Raw File Name (*.eeg)	Remark
P16	V01	D01	S01	4-Mar-19	Training	p16_v01_d01_b0001_tr01	
		D01	S02	4-Mar-19	Training	p16_v01_d01_b0002_fb01	
		D01	S03	4-Mar-19	Training	p16_v01_d01_b0003_fb02	
		D01	S04	4-Mar-19	Training	p16_v01_d01_b0004_fb03	
		D01	S05	4-Mar-19	Feedback	p16_v01_d01_b0005_fb04	
		D02	S01	5-Mar-19	*		
		D02	S02	5-Mar-19	Feedback	p16_v01_d01_b0002_fb02	
		D02	S03	5-Mar-19	Feedback	p16_v01_d01_b0003_fb03	
		D02	S04	5-Mar-19	Feedback	p16_v01_d01_b0004_fb04	
		D02	S05	5-Mar-19	*		
		D02	S06	5-Mar-19	*		
		D02	S07	5-Mar-19	Speller	p16_v01_d01_b0007_sp03	34 trials (trigger 15 and triggers of interrupted trial)
		D02	S08	5-Mar-19	Speller	p16_v01_d01_b0008_sp04	62 trials (w/o trigger 15)
		D03	S01	6-Mar-19	Training	p16_v01_d03_b0001_fb01	
		D03	S02	6-Mar-19	Feedback	p16_v01_d03_b0002_fb02	
		D03	S03	6-Mar-19	Feedback	p16_v01_d03_b0003_fb03	
		D03	S04	6-Mar-19	Feedback	p16_v01_d03_b0004_fb04	
		D03	S05	6-Mar-19	*		
		D03	S06	6-Mar-19	*		
		D03	S07	6-Mar-19	Training	p16_v01_d03_b0007_tr01	
		D03	S08	6-Mar-19	Feedback	p16_v01_d03_b0008_fb05	Only ten questions
		D03	S09	6-Mar-19	Training	p16_v01_d03_b0009_fb06	
		D03	S10	6-Mar-19	Training	p16_v01_d03_b0010_fb07	
		D04	S01	7-Mar-19	Training	p16_v01_d04_b0001_tr01	
		D04	S02	7-Mar-19	Training	p16_v01_d04_b0002_tr02	
		D04	S03	7-Mar-19	Training	p16_v01_d04_b0003_tr03	
		D04	S04	7-Mar-19	Training	p16_v01_d04_b0004_tr04	
		D04	S05	7-Mar-19	Feedback	p16_v01_d04_b0005_fb03	
		D04	S06	7-Mar-19	Feedback	p16_v01_d04_b0006_fb04	
		D04	S07	7-Mar-19	Speller	p16_v01_d04_b0006_sp01	
		D04	S08	7-Mar-19	*		
		D05	S01	8-Mar-19	Training	p16_v01_d05_b0001_tr01	
		D05	S02	8-Mar-19	Training	p16_v01_d05_b0002_tr02	
		D05	S03	8-Mar-19	Training	p16_v01_d05_b0003_tr03	
		D05	S04	8-Mar-19	Feedback	p16_v01_d05_b0003_fb02	
		D05	S05	8-Mar-19	Speller	p16_v01_d05_b0004_sp01	
		D05	S06	8-Mar-19	Speller	p16_v01_d05_b0005_sp02	
	V02	D01	S01	20-May-19	Training	p06_v02_d0001_b01_tr01	
		D01	S02	20-May-19	Training	p06_v02_d0001_b02_tr02	
		D01	S03	20-May-19	Training	p06_v02_d0001_b03_tr03	
		D01	S04	20-May-19	Training	p06_v02_d0001_b04_tr04	
		D01	S05	20-May-19	Training	p06_v02_d0001_b05_tr05	
		D01	S06	20-May-19	Training	p06_v02_d0001_b06_tr06	
		D01	S07	20-May-19	Training	p06_v02_d0001_b07_tr07	
		D02	S01	21-May-19	Training	p06_v02_d02_b00001_tr01	
		D02	S02	21-May-19	Training	p06_v02_d02_b00002_tr02	
		D02	S03	21-May-19	Feedback	p06_v02_d02_b00003_fb01	
		D02	S04	21-May-19	Feedback	p06_v02_d02_b00004_fb02	
		D02	S05	21-May-19	Feedback	p06_v02_d02_b00005_fb03	
		D02	S06	21-May-19	Speller	p06_v02_d02_b00005_sp01	
		D03	S01	22-May-19	*		
		D03	S02	22-May-19	Training	p06_v02_d03_b00002_tr02	
		D03	S03	22-May-19	Feedback	p06_v02_d03_b00003_fb01	
		D03	S04	22-May-19	Feedback	p06_v02_d03_b00004_fb02	
		D03	S05	22-May-19	Speller	p06_v02_d03_b00005_sp01	
		D04	S01	23-May-19	Training	p06_v02_d04_b00001_tr01	
		D04	S02	23-May-19	Training	p06_v02_d04_b00002_tr02	
		D04	S03	23-May-19	Feedback	p06_v02_d04_b00003_fb01	
		D04	S04	23-May-19	Feedback	p06_v02_d04_b00004_fb02	
		D04	S05	23-May-19	Feedback	p06_v02_d04_b00005_fb03	
		D04	S06	23-May-19	Feedback	p06_v02_d04_b00006_fb04	
		D04	S07	23-May-19	Feedback	p06_v02_d04_b00007_fb05	
		D04	S08	23-May-19	Feedback	p06_v02_d04_b00008_fb06	
		D04	S09	23-May-19	Speller	p06_v02_d04_b00009_fb07	

Supplementary Table S7:
Session-Raw File Correspondence for P16, Sequence of the training, feedback and spelling sessions.
A detailed list of the sequence of visits (V), days (D), sessions (S), the dates in which the session of the study was recorded from P16. The sixth column from left to right shows the type of session. Either Training, Feedback or Speller. Sessions indicated with (*) were recorded for a study outside the scope of this research or are defected recordings. Columns seven specifies the exact name of the raw file containing the recordings. Column eight gives some remarks of irregularities during the in situ recording of the raw file of the session.
